# PD-L1 chimeric costimulatory receptor improves the efficacy of CAR-T cells for PD-L1-positive solid tumors and reduces toxicity in vivo

**DOI:** 10.1186/s40364-020-00237-w

**Published:** 2020-11-02

**Authors:** Qibin Liao, Yunyu Mao, Huan He, Xiangqing Ding, Xiaoyan Zhang, Jianqing Xu

**Affiliations:** Shanghai Public Health Clinical Center & Institutes of Biomedical Sciences, Fudan University, Shanghai, China

**Keywords:** Chimeric antigen receptor, Chimeric costimulatory receptor, PD-L1, Safety, Efficacy

## Abstract

**Background:**

On-target off-tumor toxicity impedes the clinical application of chimeric antigen receptor-modified T cells (CAR-T cells) in the treatment of solid tumors. Previous reports proved that the combinatorial antigen recognition strategy could improve the safety profile of CAR-T cells by targeting two different tumor-associated antigens (TAAs), one as a CAR-T targeted antigen and the other as a chimeric costimulatory receptor (CCR) ligand. The programmed death-ligand 1 (PD-L1, also known as B7-H1) is preferentially overexpressed on multiple tumors, it will be highly interesting to explore the potential of PD-L1 as a universal target for designing CCR.

**Methods:**

A novel dual-targeted CAR, which is composed of first-generation CD19/HER2 CAR with CD3ζ signaling domain and PD-L1 CCR containing the CD28 costimulatory domain, was constructed and delivered into T cells by pseudotyped lentivirus. The cytokine release, cytotoxicity and proliferation of dual-targeted CAR-T cells were tested in vitro, and their safety and therapeutic efficacy were evaluated using a human tumor xenograft mouse model in vivo.

**Results:**

The dual-targeted CAR-T cells exerted a similar cytotoxic activity against CD19/HER2^+^ tumor cells with or without PD-L1 in vitro, however, enhanced cytokine releases and improved proliferative capacity were only observed in the presence of both CD19/HER2 and PD-L1. Importantly, the dual-targeted CAR-T cells displayed no cytotoxicity against PD-L1^+^ cells alone in the absence of tumor antigen CD19/HER2. In addition, the dual-targeted CAR-T cells preferably destroyed tumor xenografts bearing both CD19/HER2 and PD-L1*,* but spared only antigen-positive tumor xenografts without PD-L1 in vivo. Furthermore, PD-L1 CCR also improved the antitumor efficacy of the low-affinity HER2 CAR-T cells against PD-L1^+^ tumors expressing high levels of HER2.

**Conclusion:**

Our observations demonstrated that PD-L1 could be used as a universal target antigen for designing CCR, and the dual-targeted CAR-T cells equipped with PD-L1 CCR could be used to reduce the risk of on-target off-tumor toxicity while retaining their potent antitumor efficacy in the treatment of PD-L1^+^ solid tumors.

## Introduction

The CD19 CAR-T cells have achieved great success in treating hematological malignancies with higher response rates [1–3]. However, several clinical trials have shown that B cell aplasia often occurs in patients treated with CD19 CAR-T cells because of the high expression of CD19 on normal B cells [1, 4, 5]. The effect of on-target off-tumor toxicity, severe liver toxicity, was also observed in the treatment of renal cell carcinoma using CAR-T cells targeting carbonic anhydrase IX (CAIX) due to the expression of CAIX on normal bile duct epithelial cells [6]. In addition, human epidermal growth factor receptor 2 (HER2)-targeted CAR-T cells led to a lethal cytokine storm, which was attributed to the recognition of low levels of HER2 on lung epithelial cells [7]. Therefore, it is critical to reduce the on-target off-tumor toxicity of CAR-T cells but retain their efficacy.

Synthetic biology approaches were developed to attenuate on-target off-tumor toxicity of CAR-T cells, such as CTLA-4/PD-1-based inhibitory CAR system, or a CAR and chimeric costimulatory receptor (CCR)-based AND gate strategy: the CAR provides the first signal for the suboptimal activation after encountering a TAA, and the CCR engages with another TAA to provide a costimulatory signal for optimal activation [8]. The masked CAR-T cells could selectively kill cancer cells within the matrix metalloproteinase (MMP)-enriched tumor microenvironment as MMP degrades the mask to expose the CAR [9]. The synNotch receptor-engineered T cells apply a synthetic Notch receptor to recognize a TAA and then trigger the expression of a CAR that binds to another TAA [10]. Recently, a SUPRACAR system was also developed to enhance the specificity of engineered T cells [11]. Among these strategies, the feasibility of combinatorial antigen recognition approach has been confirmed in the different solid tumors, including HER2^+^MUC1^+^ breast cancer [12], PSCA^+^PSMA^+^ prostate cancer [8], GPC3^+^ASGR1^+^ hepatocellular carcinoma [13] and CEA^+^MSLN^+^ pancreatic cancer [14]. One concern is that the strategy may hinder the broad application of CAR-T cells because of tumor antigen heterogeneity, e.g., MSLN was found in 25 ~ 30% of breast cancers, 40 ~ 45% of colon cancers and 80 ~ 85% of pancreatic cancers [15], thus choosing a broadly expressed antigen on tumor cells to design CCR may promote the clinical application of CAR-T cells using combinatorial antigen recognition strategy.

It was reported that PD-L1/B7-H1 antigen is abundant on multiple tumors, e.g., 100% of melanomas, 95.2% of lung cancers and 90% of ovarian cancers, whereas only low expression has been identified in normal tissues [16]. Therefore, PD-L1 may be explored as a universal target for designing CCR. Moreover, tumor-associated PD-L1 inhibits the antitumor response by engaging with PD-1 expressed on T cells, and the blockade of PD-L1 or PD-1 can restore the host antitumor immune response [17]. Therefore, PD-L1 may be employed not only as a universal target for designing CCR, but also as a switch to turn the “immune brake” into “immune accelerator”.

To test this concept, we constructed a novel dual-targeted CAR composed of a TAA (CD19/HER2)-targeted first-generation CAR and a universal CCR specific for PD-L1. The functionality of the dual-targeted CAR-T cells was verified both in vitro and in vivo. We demonstrated that upon binding to PD-L1, the PD-L1 CCR provided an efficient costimulatory signal for CD19/HER2-targeted CAR-T cells to enhance the cytokine releases and proliferation in vitro. It could also potently eliminate tumor xenografts bearing both TAAs and PD-L1 but not only TAAs^+^ tumor xenografts. The results evidenced that the PD-L1 CCR is a safer and more effective therapeutic modality for PD-L1-positive malignant tumors.

## Methods

### Cell lines

The following cell lines were used: K562 (a chronic myelogenous leukemia cell line, ATCC #CCL-243), Jurkat, clone E6–1 (an acute T cell leukemia cell line, ATCC #TIB-152), A549 (a lung cancer cell line, ATCC #CCL-185), NCI-H292 (a lung cancer cell line, ATCC #CRL-1848), SKOV3 (an ovarian cancer cell line, HTB-77) and HEK293T (an embryonic kidney cell line, ATCC #CRL-3216). All cell lines were purchased from ATCC and maintained in RPMI 1640 (Corning #10–040-CVR) supplemented with 10% fetal bovine serum (FBS) (BI #04–001-1acs) and 1% penicillin-streptomycin (Corning #30–002-CI), except for HEK293T cells, which were cultured in DMEM (Corning #10–013-CV) supplemented with 10% FBS and 1% penicillin-streptomycin. All tumor cell lines used in our study expressed little or no PD-L1 (Additional file 2: Figure S2). Thus, pseudotyped viruses carrying the PD-L1 gene were delivered into tumor cells to produce lines that had high, stable expression of PD-L1, e.g., K562-PD-L1, A549-PD-L1, NCI-H292-PD-L1 and SKOV3-PD-L1. Furthermore, the firefly luciferase gene or CD19 was also delivered into tumor cells via pseudotyped lentiviruses to generate A549-Luc, A549-Luc-PD-L1, A549-Luc-CD19, A549-Luc-CD19-PD-L1, NCI-H292-Luc-CD19, NCI-H292-Luc-CD19-PD-L1, SKOV3-Luc and SKOV3-Luc-PD-L1. These cells were maintained in a humidified atmosphere containing 5% CO_2_ at 37 °C.

### CAR and CCR designs

**Fig. 1:**
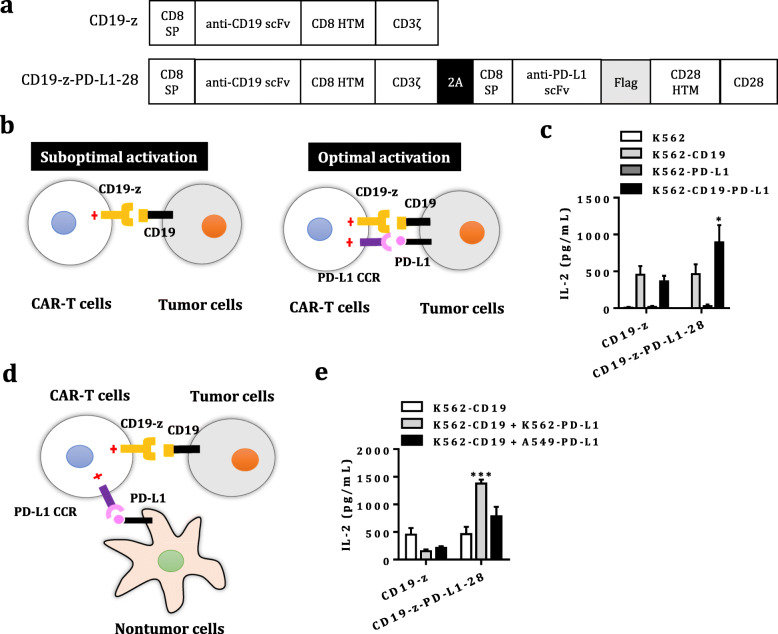
Design and characterization of the PD-L1 CCR in Jurkat T cells. **a** Schematic representation of the CD19 CAR and PD-L1 CCR constructs. The CD19 CAR (CD19-z) was generated by using the first-generation CAR that contains the CD19-targeting scFv fused to the human CD8 hinger and transmembrane domain, followed by the CD3ζsignaling domain. CD19-z-PD-L1–28 was generated by linking CD19-z to the PD-L1 CCR that generated by fusing a humanized PD-L1-binding scFv to the hinger, transmembrane and human CD28 costimulatory domain, via the self-cleaving T2A peptide sequence. **b** The first-generation CAR-engineered T cells achieve suboptimal activation when exposed to a single target antigen but are fully activated by tumor cells expressing target antigen and PD-L1 through coexpression of the PD-L1 CCR, providing a costimulatory signal. **c** The levels of IL-2 in the supernatant secreted by CD19-z- and CD19-z-PD-L1–28-engineered Jurkat T cells in the 24 h co-culture system (E:T, 1:1) were measured by ELISA. The results are presented as the mean ± SEM of seven independent experiments, * *P* < 0.05 with respect to co-culture with CD19^+^ K562 cells, analyzed using Student’s t-test. **d** Dual-targeted CAR-T cells can be stimulated with PD-L1^+^ host cells (e.g., macrophages, dendritic cells) when exposed to PD-L^−^tumor cells expressing CAR-targeted antigen. **e** The levels of IL-2 released by CD19-z and CD19-z-PD-L1–28-engineered Jurkat T cells were measured by ELISA after 24 h of incubation at an E:T:host cell ratio of 3:1:1. The results are reported as the mean ± SEM of three independent experiments, *** *P* < 0.001 with respect to co-culture with CD19^+^ K562 cells, analyzed using Student’s t-test

DNA encoding the human CD8 signal peptide (SP) (NP_001759.3 aa 1–21), human PD-L1-binding scFv, Flag tag (DYKDDDDK), the hinger spacer, transmembrane and signaling domain of human CD28 (NP_006130.1 aa 114–220), the human 4-1BB signaling domain (AAA53133.1 aa 209–255) and the human CD3ζ cytosolic domain (NP_932170 aa 52–164) was cloned into the empty lentiviral transfer plasmid (pHAGE_EF1α_MCS_IRES_ZsGreen) to generate the recombinant lentiviral transfer plasmid containing PD-L1 CAR (pHAGE_PD-L1–28BBz) (Additional file 1: Figure S1a). The expression cassette encoding human CD8 SP, anti-human CD19 scFv (FMC63 clone), human HER2 scFv (4D5 clone) or low-affinity HER2 scFv (4D5–5 clone), Myc tag (EQKLISEEDL), the hinger and transmembrane domain of CD8 (NP_001759.3 aa 138–206) and the CD3ζ cytosolic domain was cloned into another empty lentiviral transfer plasmid (pKL_EF1α_MCS_P2A_EGFP) to generate recombinant lentiviral transfer plasmids carrying CD19 CAR (pKL_CD19-z) or HER2 CAR (pKL_HER2-z, pKL_La-HER2-z) (Fig. 1a and Additional file 3: Figure S3). The artificial gene encoding both the CD19/HER2 CAR and the PD-L1 CCR is composed of the CD8 SP, PD-L1 scFv, Flag tag, and the hinger, transmembrane and signaling domain of CD28 (PD-L1–28). The CD19/HER2 CAR was linked to the PD-L1 CCR via the self-cleaving T2A peptide sequence. The expression cassette encoding both the CAR and CCR was also cloned into the empty pKL_EF1α_MCS_P2A_EGFP plasmid to generate the recombinant pKL_CD19-z-PD-L1–28 and pKL_HER2-z-PD-L1–28 plasmids (Fig. 1a and Additional file 3: Figure S3). All genes were synthesized by Generay Biotech (Shanghai) Co., Ltd. The lentiviral transfer plasmids also encoded enhanced green fluorescent protein (EGFP) or ZsGreen to evaluate the transduction efficiency of pseudoviruses carrying the CAR or CCR.

### Lentivirus production

Transient lentiviral supernatant was produced as described below. Lentiviral vectors were prepared by transient transfection of HEK293T cells using TurboFect transfection reagent (Thermo Scientific #R0531). HEK293T cells (6 × 10^6^) cultured in 10-cm tissue culture dishes were transfected with the lentiviral transfer plasmid (3 μg), the VSV-G envelope plasmid PMD2.G (Addgene #12259) (3 μg) and the packaging plasmid psPAX2 encoding gag-pol (Addgene #12260) (9 μg). The lentiviral supernatant was harvested 48 h after transfection and filtered through a 0.45-μm filter (PALL #4614). Lentiviral particles were further concentrated by ultracentrifugation for 2 h at 28000 rpm with a Beckman SW28 rotor (Beckman) before use.

### T cells transduction and expansion

Frozen human peripheral blood mononuclear cells (PBMCs) were obtained from Shanghai Public Health Clinical Center. PBMCs were thawed in T cell growth medium (TCM), consisting of X-VIVO 15 medium (Lonza #BE02-060F), human IL-7 (R&D systems #P13232), human IL-15 (R&D systems #P40933) and human IL-21 (Novoprotein #GMP-CC45), and then rested for 4 ~ 6 h. The thawed PBMCs were further sorted into CD4^+^ and CD8^+^ T cells with an EasySep™ Human CD4^+^ T Cell Enrichment Kit (STEMCELLS #19052) and EasySep™ Human CD8^+^ T Cell Enrichment Kit (STEMCELLS #19053), respectively. Before transduction, PBMCs or sorted CD4^+^/CD8^+^ T cells were stimulated for 24 ~ 36 h with anti-hCD3 and anti-hCD28-coated immunobeads at a bead-to-cell ratio of 1:1 in TCM. The activated T cells were incubated with freshly concentrated lentiviral vectors in NovoNectin (Novoprotein # GMP-CH38)-coated 48-well flat plates at 32 °C and centrifuged at 1000×g for 1.5 h. The culture medium was changed to fresh TCM overnight. The immunobeads were removed 6 ~ 7 days post-transduction, and the T cells were expanded until they were rested and could be used in further assays. During ex vivo expansion, the TCM was replenished, and the cell density was adjusted to 0.5 ~ 2 × 10^6^/mL every 3 days.

### Generation of CAR-engineered Jurkat T cells

Jurkat T cells were transduced with lentiviral particles carrying PD-L1–28BBz, CD19-z or CD19-z-PD-L1–28. Jurkat T cells expressing EGFP/ZsGreen were sorted by flow cytometry (BD FACS Aria II). The sorted Jurkat T cells were expanded and then used in further assays.

### Cell aggregation assay

To determine whether the PD-L1 CAR-engineered Jurkat T cells bound to PD-L1 presented on the tumor cell surface to promote cell aggregation, PD-L1-positive K562 tumor cells labeled with Cell Proliferation Dye eFluor 670 (Invitrogen #65–0840) were mixed with PD-L1 CAR-modified Jurkat T cells or untransduced Jurkat T cells labeled with CellTrace CFSE (Invitrogen #C34554) at an effector: target (E: T) ratio of 1:1 in a 1.5 mL eppendorf tube at room temperature (RT) for 1 h. The proportion of cells forming heterologous cell aggregates (eFluor 670^+^CFSE^+^) was assessed by flow cytometry (BD LSRFortessa).

### Surface immunostaining and flow cytometry

For tumor cells expressing CD19 and/or PD-L1, 5 × 10^5^ tumor cells were harvested and washed twice with FACS buffer (1× PBS containing 2% FBS). Then, tumor cells were stained with 0.5 μL of APC/Cy7-conjugated mouse anti-human CD19 (BD Pharmingen #557791) or 0.5 μL of APC-conjugated mouse anti-human PD-L1 (eBioscience #17–5983-73) at 4 °C for 30 min, washed with FACS buffer twice, and resuspended in FACS buffer for assessment. Additionally, 5 × 10^5^ washed tumor cells were incubated with 2 μg/mL of trastuzumab prepared in our laboratory at 4 °C for 30 min, washed twice with FACS buffer, and further stained with 0.5 μL of PE-conjugated anti-human IgG Fc (Biolegend #409304) at 4 °C for 30 min, washed with FACS buffer twice, and then resuspended in FACS buffer to detect HER2. To detect the HER2 CAR, PD-L1 CAR or PD-L1 CCR presented on the T cells surface, T cells were stained with Alexa Fluor 647-conjugated anti-Myc tag (CST #2233S) or PE-conjugated anti-DYKDDDDK (Biolegend #637310). T cells (5 × 10^5^) were harvested and washed twice with FACS buffer. For Myc or Flag tag staining, T cells were stained with 0.5 μL of Alexa Fluor 647-conjugated anti-Myc tag or PE-anti-DYKDDDDK at 4 °C for 30 min, washed twice with FACS buffer, and then resuspended in FACS buffer for detection. Fluorescence was assayed using a BD LSRFortessa, and all FACS data were analyzed with FlowJo V10 software.

### In vitro stimulation of CAR-T cells and quantitation of cytokines

CAR-T cells (1 × 10^5^) were co-cultured with K562 cells at an effector: target (E:T) ratio of 1:1. After mixing CAR-T cells and K562 cells in a 96-well round-bottom plate, the plate was centrifuged for 1 min at 400×g to force cell interactions. For adherent tumor cells, 1 × 10^5^ CAR-T cells were co-cultured with tumor cells at an E:T ratio of 3:1 in a 96-well flat-bottom plate. The co-culture supernatant was collected after 24 h and stored at − 20 °C for further quantitation. IL-2, IFN-γ or TNF-α levels in the culture supernatant were determined using the Human IL-2 ELISA Set (BD Biosciences #555190), Human IFN-γ ELISA Set (BD Biosciences #555142) or BD CBA Human Th1/Th2/Th17 Cytokine Kit (BD Biosciences #560484).

### In vitro evaluation of CAR-T cells cytotoxicity

Tumor cell lysis by CAR-T cells was assessed by using an 18-h luciferase-based killing assay. Briefly, tumor cells expressing target antigen and luciferase were plated in triplicate in a 96-well black flat-bottom plate (Greiner #655090) at a density of 1 × 10^4^ cells per well and grown for 18 ~ 24 h. CAR-T cells were added to the plate at E:T ratios of 0.5:1, 1:1, 2:1 and 4:1 and then cultured for another 18 h. The culture supernatant was removed, and the viability of the tumor cells was assessed by quantifying the firefly luciferase fluorescence intensity with a GloMax® 96 reader (Promega #E6521) using a Luciferase Assay System (Promega #E1501). The formula used to calculate the percent normalized cytotoxicity is as follows:

$$ 100\%-\left(\mathrm{luciferase}\ \mathrm{fluorescence}\ \mathrm{in}\mathrm{tensity}\ \mathrm{in}\ \mathrm{untransduced}\ \mathrm{T}\ \mathrm{cells}\ \mathrm{well}-\mathrm{luciferase}\ \mathrm{fluorescence}\ \mathrm{in}\mathrm{tensity}\ \mathrm{in}\ \mathrm{assay}\ \mathrm{well}\right)/\left(\mathrm{luciferase}\ \mathrm{fluorescence}\ \mathrm{in}\mathrm{tensity}\ \mathrm{in}\ \mathrm{untransduced}\ \mathrm{T}\ \mathrm{cells}\ \mathrm{well}\right). $$

A flow cytometry-based assay was also used to measure cytotoxicity for CAR-T cells co-cultured with multiple cell lines (e.g. K562-PD-L1, A549, NCI-H292, and SKOV3). Briefly, CAR-T cells were co-incubated at different ratios (e.g. 1:1, 2:1 and 4:1) with pre-labeled adherent tumor cells (e.g. A549, NCI-H292, and SKOV3) or K562-PD-L1 cells. The adherent tumor cells were labeled with Cell Proliferation Dye eFluor 450 (Invitrogen #65–0842-85), and K562-PD-L1 cells were labeled with Cell Proliferation Dye eFluor 670 at a final concentration of 5 μM to allow their discrimination from CAR-T cells. After 12 h, dead target cells were identified by propidium iodide (PI) staining (BD #556547) using flow cytometry. The formula used to calculate the percent normalized cytotoxicity is as follows:

$$ 100\%-\left(100\%-\%\mathrm{of}\ \mathrm{dead}\ \mathrm{cells}\ \mathrm{in}\ \mathrm{assay}\ \mathrm{well}\right)/\left(100\%-\%\mathrm{of}\ \mathrm{dead}\ \mathrm{cells}\ \mathrm{in}\ \mathrm{untransduced}\ \mathrm{T}\ \mathrm{cells}\ \mathrm{well}\right). $$

### Detection of the in vitro proliferation of CAR-T cells

For in vitro proliferation assays, single-targeted and dual-targeted CAR-T cells were washed with 1× PBS and then labeled with Cell Proliferation Dye eFluor 670 at a final concentration of 5 μM, according to the manufacturer’s instructions. Tumor cells expressing CD19/HER2 or PD-L1 were treated with 50 μg/mL mitomycin C (Biotech well #WF0197) to result in replication-defective tumor cells. Then, these pre-labeled CAR-T cells were co-cultured with pretreated tumor cells at an E:T ratio of 2:1 in RPMI 1640 supplemented with 10% FBS and 1% penicillin-streptomycin, and the mixed cells were collected for flow cytometry analysis. Finally, the proliferation of CAR-T cells was assayed by monitoring the dilution of the cell proliferation dye after 4 days of co-culture.

### Xenograft dual-tumor models

The animal protocols used in this study were approved by the Institutional Animal Care and Use Committee (IACUC) of Shanghai Public Health Clinical Center. Female NOD-*Prkdc*^*scid*^ *Il2rg*^*tm1*^/Bcgen (B-NDG) (Biocytogen) mice aged 6 ~ 8 weeks were subcutaneously inoculated with four xenograft tumors in the right flank: 1) 5 × 10^6^ CD19^+^ and 5 × 10^6^ CD19^+^PD-L1^+^ A549 cells; 2) 2 × 10^6^ CD19^+^ and 2 × 10^6^ CD19^+^PD-L1^+^ NCI-H292 cells; 3) 2 × 10^6^ HER2^+^ and 2 × 10^6^ HER2^+^PD-L1^+^ NCI-H292 tumor cells; or 4) 1 × 10^6^ HER2^+^ and 1 × 10^6^ HER2^+^PD-L1^+^ SKOV3 cells. Five or 10 days after tumor inoculation, 2 × 10^6^ CD4^+^ and CD8^+^ T cells were injected intravenously into the A549 tumor-bearing mice, while 4 × 10^6^ CD4^+^ and CD8^+^ T cells were injected into the NCI-H292/SKOV3 tumor-bearing mice. These T cells were either untransduced or engineered with single-targeted CAR or dual-targeted CAR. Tumor size was monitored by calipers every 3, 5 or 10 days after T cells transfer, and tumor volume was calculated with the following formula: V = (length × width^2^)/2. Mice were considered dead when the tumor size reached the euthanasia criteria.

### Statistical analysis

All data are presented as the mean ± standard error of the mean (SEM) unless otherwise described. Statistical differences were determined by a paired Student’s t-test (two-tailed) unless otherwise noted. The statistical significance of differences among three or more groups was analyzed by one-way ANOVA with Tukey’s test for further multiple comparisons. A *P*-value < 0.05 was considered statistically significant. All statistical analyses were performed with Prism 7.0 (GraphPad), and statistical significance was reported as *P ≤ 0.05, **P ≤ 0.01, ***P ≤ 0.001, and ****P ≤ 0.0001.

## Results

### Characterization of the PD-L1 scFv-based CAR and CCR in Jurkat T cells

The PD-L1/B7-H1 antigen is preferentially expressed in many different types of cancer cells but minimally expressed on normal tissues [16], indicating its potential as an ideal target for designing CAR-T cells to combat multiple solid tumors [18–20]. Therefore, we tested whether we can design a CAR or CCR incorporating a humanized scFv specific for PD-L1 that can be used for cell-based immunotherapy. Initially, PD-L1 overexpressing K562 and A549 tumor cells were constructed to test the functionality of PD-L1 CAR (Additional file 1: Figure S1b). Then, we generated a PD-L1 scFv by fusing a variable region of the light chain to the heavy chain via a GS linker used in a CD19 scFv-based CAR [21] based on sequences of PD-L1-neutralized antibodies (US8552154), which was used to construct PD-L1 CAR (Additional file 1: Figure S1a). To determine the activity of PD-L1 CAR, we first engineered Jurkat T cells to stably express the PD-L1 CAR (Additional file 1: Figure S1c). We observed that cell aggregates were only found in the co-culture of PD-L1 CAR-Jurkat T cells and K562-PD-L1 cells (6.52%), but none in the co-culture of un-transduced Jurkat T cells and K562-PD-L1 cells (Additional file 1: Figure S1d). It was known that Jurkat T cells can produce a large amount of IL-2 after T cells receptor (TCR) activation [22]. As expected, upon stimulation with PD-L1 on tumor cells, PD-L1 CAR-Jurkat T cells produced high levels of IL-2 (Additional file 1: Figure S1e), which indicated the binding of this anti-PD-L1 scFv to PD-L1 is capable of transducing signal into T cells and thereby applicable in designing CCR.

The combinatorial antigen recognition strategy was proved to reduce on-target off-tumor toxicity of CAR-T cells in solid tumors. Since PD-L1 is usually overexpressed in tumor microenvironment, we rationalized that PD-L1 could be used as a universal target for designing a CCR to mitigate toxicity of CAR-T cells. To prove this concept, we employed CD19 CAR as a model. The first-generation CD19 CAR was generated to provide the initial suboptimal signal for T cells activation, and PD-L1 CCR containing CD28 costimulatory domain for optimal activation (Fig. 1a-b). The dual-targeted CAR-Jurkat T cells stimulated by CD19^+^PD-L1^+^ K562 cells produced a higher level of IL-2 than that of CD19^+^ K562 cells, whereas comparable levels of IL-2 in CD19 CAR-Jurkat T cells stimulated by CD19^+^ K562 and CD19^+^PD-L1^+^ K562 cells (Fig. 1c).

Recent studies have proven that PD-L1 on host cells is critical for PD-L1 blockade therapy, regardless of whether tumor cells express PD-L1 [23, 24]. Therefore, we hypothesized that PD-L1 expressed on non-tumor cells in the tumor microenvironment, including bone marrow-derived cells and stromal cells, may also trigger the PD-L1 CCR presented on CAR-T cells to provide costimulatory signal for optimal activation (Fig. 1d). Indeed, the addition of K562-PD-L1 or A549-PD-L1 to the co-culture of dual-targeted-Jurkat T cells and K562-CD19 or A549-CD19 cells also enhanced the production of IL-2 (Fig. 1e).

### Enhanced cytokine releases of dual-targeted CAR-T cells equipped with PD-L1 CCR

**Fig. 2:**
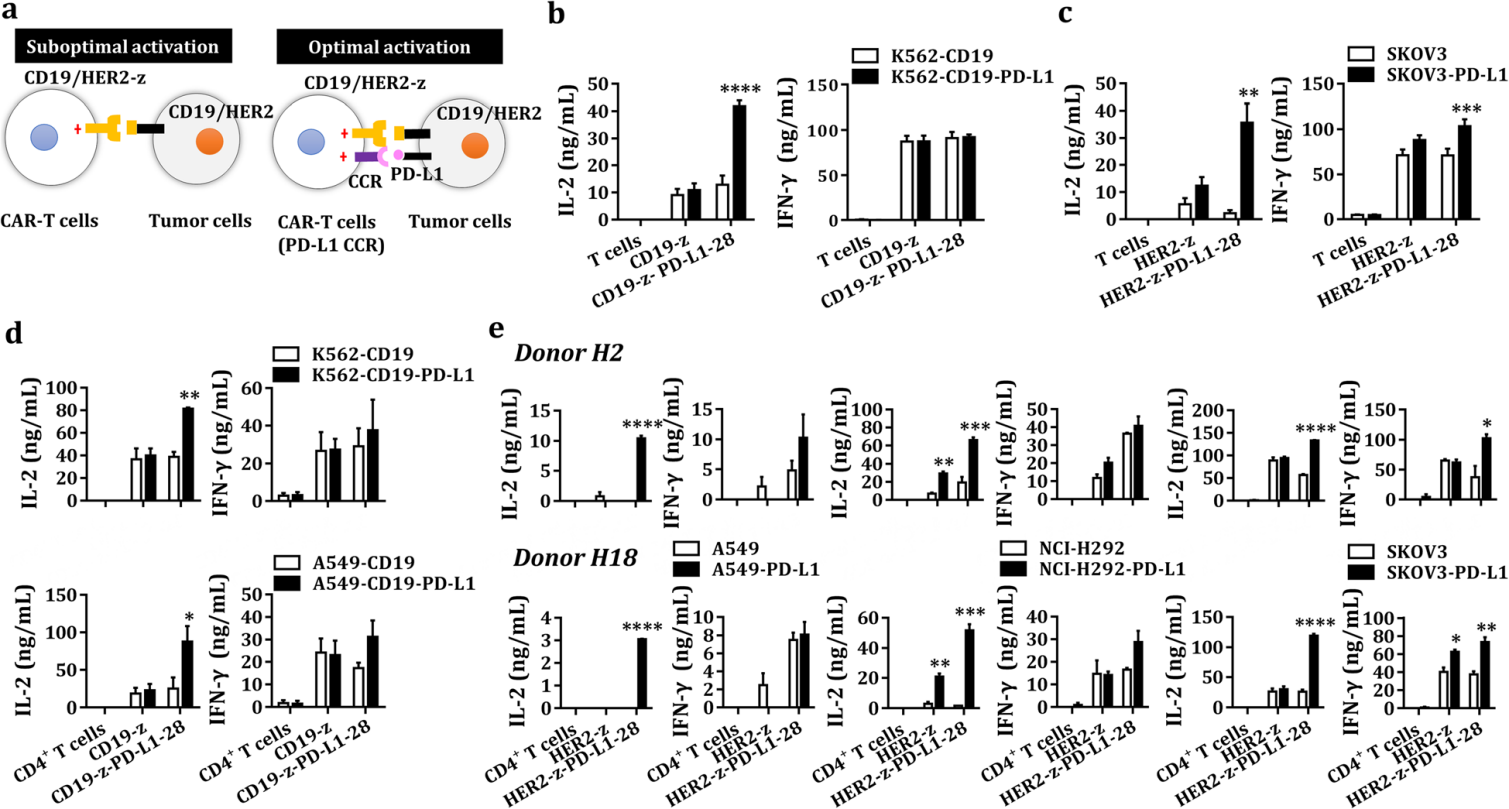
Characterization of the PD-L1 CCR in primary T cells. **a** CD19/HER2-z-expressing T cells received a suboptimal activation signal when exposed to a single antigen, but dual-targeted CAR-T cells achieved optimal activation when engaged with CD19/HER2 and PD-L1. **b-c** The levels of IL-2 and IFN-γ produced by untransduced, CD19-z or CD19-z-PD-L1–28-engineered T cells (**b**) and HER2-z or HER2-z-PD-L1–28-engineered T cells (**c**) were measured by ELISA after 24 h of coincubation at an E:T ratio of 1:1 (**b**) or 3:1 (**c**). The results are reported as the mean ± SEM for five healthy donors (**b**) or three healthy donors (**c**), ** *P* < 0.01, *** *P* < 0.001, **** *P* < 0.0001 with respect to co-culture with CD19^+^ K562 cells (**b**) or SKOV3 cells (**c**), analyzed using a paired Student’s t-test. **d** The levels of IL-2 and IFN-γ secreted by untransduced, CD19-z- or CD19-z-PD-L1–28-engineered CD4^+^ T cells were measured by ELISA after 24 h incubation at an E:T ratio of 1:1 (K562) or 3:1 (A549). The results are reported as the mean ± SEM for three healthy donors, * *P* < 0.05, ** *P* < 0.01 with respect to co-culture with CD19^+^ K562/A549 cells, analyzed using a paired Student’s t-test. **e** The levels of IL-2 and IFN-γ produced by untransduced, HER2-z or HER2-z-PD-L1–28-engineered CD4^+^ T cells were measured by ELISA after 24 h incubation at an E:T ratio of 3:1. The results are reported as the mean ± SEM for two healthy donors with technical triplicates, * *P* < 0.05, ** *P* < 0.01, *** *P* < 0.001, **** *P* < 0.0001 with respect to co-culture with A549/NCI-H292/SKOV3 cells, analyzed using Student’s t-test

We next tested whether the PD-L1 CCR works in primary T cells. To verify whether the PD-L1 CCR is active in primary T cells, dual-targeted CAR-T cells equipped with PD-L1 CCR were stimulated with K562-CD19 cells expressing with/without PD-L1 (Fig. 2a). Only dual-targeted CAR-T cells showed improved activation and presented high levels of IL-2 when exposed to CD19^+^PD-L1^+^ K562 cells (Fig. 2b). Considering that CD4^+^ T cells are the major T cell subsets that produces IL-2, we further tested the functionality of the PD-L1 CCR in primary CD4^+^ T cells. As expected, dual-targeted CAR-CD4^+^ T cells produced higher levels of IL-2 in co-cultures with CD19^+^PD-L1^+^ K562/A549 cells than that in co-cultures with CD19^+^ K562/A549 cells (Fig. 2d). Additionally, the production of IFN-γ was higher in dual-targeted CAR-CD4+ T cells activated by CD19^+^PD-L1^+^ K562/A549 cells than that in CD19^+^ K562/A549 cells, though the difference did not reach statistical significance (Fig. 2d).

Considering that CD19 itself is not expressed in K562/A549 cells, we next investigated a TAA, HER2/ERBB2, which is overexpressed in various solid tumors (Additional file 2: Figure S2), to confirm the reliability and versatility of the PD-L1 CCR. We first constructed the first-generation HER2 CAR and PD-L1 CCR expressing T cells, including CD4^+^ and CD8^+^ T cells (Additional file 3: Figure S3b), then demonstrated that only dual-targeted CAR-T cells produced high levels of IL-2 and IFN-γ when exposed to PD-L1^+^ SKOV3 cells (Fig. 2c). Similarly, dual-targeted CAR-T cells -CD4^+^ T cells secreted higher levels of IL-2 in the co-cultures of PD-L1^+^ tumor cells (e.g. A549, NCI-H292, SKOV3) than that in the co-cultures of HER2^+^ tumor cells (Fig. 2e). As described above, the production of IFN-γ was also higher in dual-targeted CAR-CD4^+^ T cells activated by PD-L1^+^ SKOV3 cells than that in SKOV3 cells (Fig. 2e).

### PD-L1 on host normal cells triggers PD-L1 CCR

**Fig. 3:**
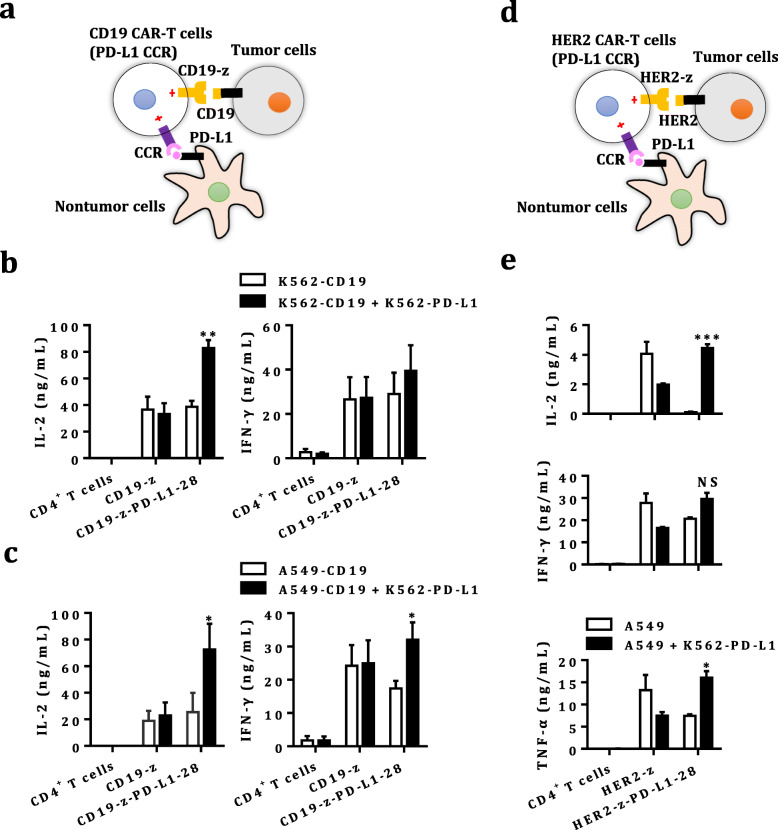
The impact of host cells PD-L1 on dual-targeted CAR-T cells. **a-c** The levels of IL-2 and IFN-γ released by untransduced, CD19-z and CD19-z-PD-L1–28-engineered CD4^+^ T cells were measured by ELISA after 24 h incubation at an E:T:host cell ratio of 1:1:1 (**b**, K562) or 3:1:1 (**c**, A549). The results are reported as the mean ± SEM for three healthy donors, * *P* < 0.05, ** *P* < 0.01 with respect to co-culture with CD19^+^ K562 (**b**) or A549 (**c**) cells, analyzed using a paired Student’s t-test. **d-e** The levels of IL-2, IFN-γ, and TNF-α secreted by untransduced, HER2-z and HER2-z-PD-L1–28-engineered CD4^+^ T cells were measured by CBA after 24 h incubation at an E:T:host cell ratio of 3:1:1. The results are reported as the mean ± SEM with technical triplicates, NS, no significance, * *P* < 0.05, *** *P* < 0.001 with respect to co-culture with A549 cells, analyzed using Student’s t-test

We also tested whether PD-L1 expressed on host cells could trigger the activation of PD-L1 CCR on CAR-T cells in the presence of cognate antigens (Fig. 3a & Fig. 3d). PD-L1 presented on K562 cells, which mimicked host normal cells, increased the production of IL-2 and IFN-γ in the co-cultures of dual-targeted CAR-CD4^+^ T cells and CD19^+^ K562 (Fig. 3b) or CD19^+^A549 cells (Fig. 3c). Similarly, K562-PD-L1 cells also enhanced the production of IL-2, IFN-γ and TNF-α in co-cultures of dual targeted CAR-CD4^+^ T cells and HER2^+^A549 cells in the context of HER2 CAR (Fig. 3e).

### Increased proliferation potential of dual-targeted CAR-T cells equipped with PD-L1 CCR

**Fig. 4:**
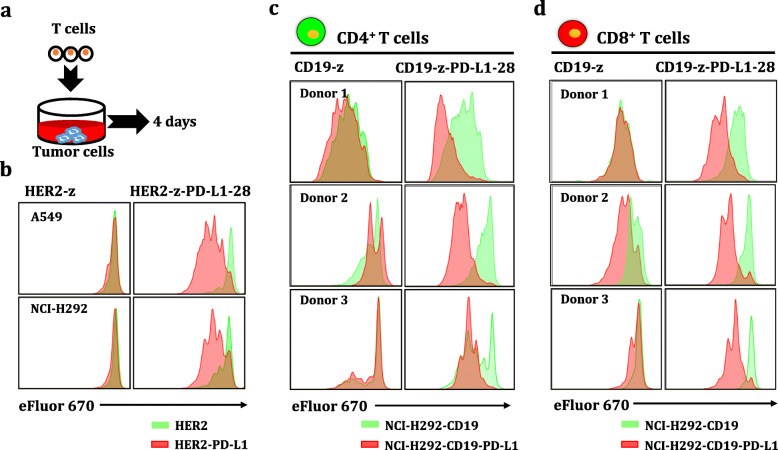
Combinatorial antigen-dependent engineered T cells proliferation. **a** Scheme of in vitro CAR-T cells proliferation. CD19/HER2 CAR-, dual-targeted CAR-T cells equipped with PD-L1 CCR and untransduced T cells were pre-labeled with Cell Proliferation Dye eFluor 670 according to the manufacturer’s instructions. A549, NCI-H292, and CD19^+^NCI-H292 cells expressing PD-L1 or not were pretreated with mitomycin C to obtain replication-incompetent target cells. These pre-labeled T cells were co-cultured with mitomycin C-treated target cells at an E:T ratio of 2:1 in RPMI 1640 medium supplemented with 10% FBS without cytokines. Finally, the proliferation of T cells was assayed by monitoring the dilution of the cell proliferation dye after incubation for 4 days. **b** Representative histogram presenting the intensity of cell proliferation dye fluorescence of HER2 CAR-T cells equipped with or without PD-L1 CCR. **c-d** Representative histogram presenting the intensity of cell proliferation dye fluorescence of CD19 CAR-CD4^+^ (**c**) and CD8^+^ (**d**) (*n* = 3)

We next determined whether the expansion of dual-targeted CAR-T cells relied on tumor cells expressing both TAAs and PD-L1. CAR-T cells were pre-labeled with cell proliferation dye to trace T cells expansion and then added to cultures of replication-defective cancer cells (Fig. 4a). Our data showed that dual-targeted CAR-T cells had a higher proliferation capacity in the presence of PD-L1^+^ tumor cells (Fig. 4b), whereas single-targeted HER2 CAR-T cells presented lower proliferation activity. Additionally, dual-targeted CAR-CD4^+^ and CD8^+^ T cells also had a higher expansion capacity in the presence of CD19^+^PD-L1^+^ tumor cells, while single-targeted CD19 CAR-T cells presented lower proliferation potential (Fig. 4b-c). In summary, TAAs and PD-L1 are required for the optimal proliferation of dual-targeted CAR-T cells, whereas TAAs alone did not fully activate CAR-T cells into proliferation.

### Dual-targeted CAR-T cells exert cytotoxicity against TAA^+^ tumor cells but spare TAA^−^PD-L1^+^ cells in vitro

**Fig. 5:**
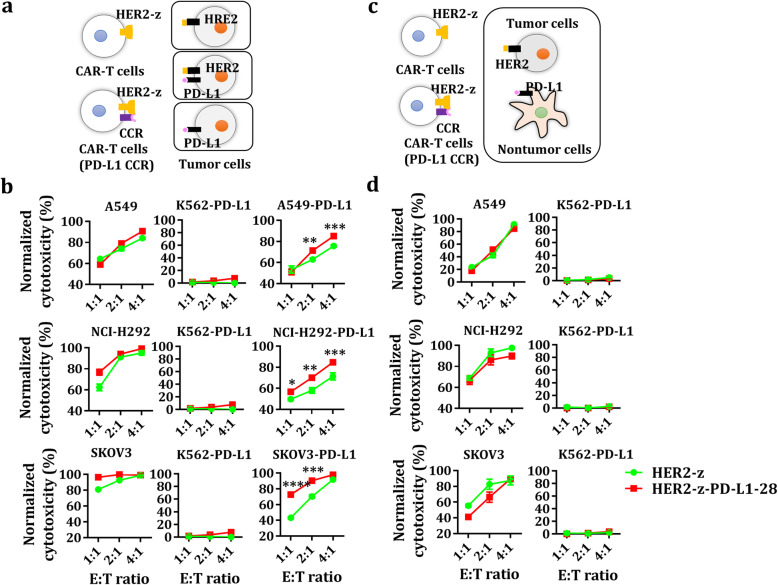
Tumor lysis of engineered T cells against cancer cells. **a-b** Untransduced, HER2-z, HER2-z-PD-L1–28-engineered T cells were used in co-culture with PD-L1^+/−^ A549, NCI-H292, SKOV3 or K562-PD-L1 cells at the indicated E:T ratios for 18 h. The results shown are the mean ± SEM for three healthy donors. * *P* < 0.05, ** *P* < 0.01, *** *P* < 0.001, **** *P* < 0.0001 with respect to co-culture with CAR-T cells without the PD-L1 CCR, analyzed using a paired Student’s t-test. **c-d** Untransduced, HER2-z, HER2-z-PD-L1–28-engineered T cells were used in co-culture with PD-L1^+^ K562 and A549, NCI-H292, or SKOV3 cells at the indicated E:T ratios for 18 h. Each value shown is the mean ± SEM of triplicates

The in vitro cytotoxic potential of dual-targeted and single-targeted CAR-CD8^+^ T cells were first examined using CD19 and/or PD-L1-overexpressed A549/NCI-H292 and K562-PD-L1 cells. Both CAR-T cells showed vigorous cytotoxicity against CD19^+^ tumor cells with or without PD-L1 but not against CD19^−^PD-L1^+^ cells (Additional file 4: Figure S4a-b). Similarly, both dual-targeted CAR-T cells and single-targeted CAR-T cells also showed active cytotoxicity against HER2^+^ tumor cells with or without PD-L1 but not against HER2^−^PD-L1^+^ cells. Interestingly, the PD-L1 CCR could improve the cytotoxic activity of dual-targeted CAR-T cells against HER2^+^PD-L1^+^ tumor cells compared to that of single-targeted HER2 CAR-T cells which was attenuated in the presence of PD-L1 on HER2^+^ tumor cells (Fig. 5a-b), indicating that the PD-L1 CCR could partially antagonize PD-L1 mediated inhibition.

Additionally, we also determined if normal cells expressing PD-L1 alone could be lysed by dual-targeted CAR-T cells when co-cultured with tumor cells. As shown, dual-targeted CAR-T cells exerted cytotoxicity against CD19^+^/HER2^+^ tumor cells, but spared PD-L1^+^ normal cells in the context of either CD19 (Additional file 4: Figure S4c-d) or HER2 CAR (Fig. 5c-d).

### Dual-targeted CAR-T cells exhibit vigorous antitumor activity against PD-L1^+^ tumor xenografts but not PD-L1^−^ xenografts

**Fig. 6:**
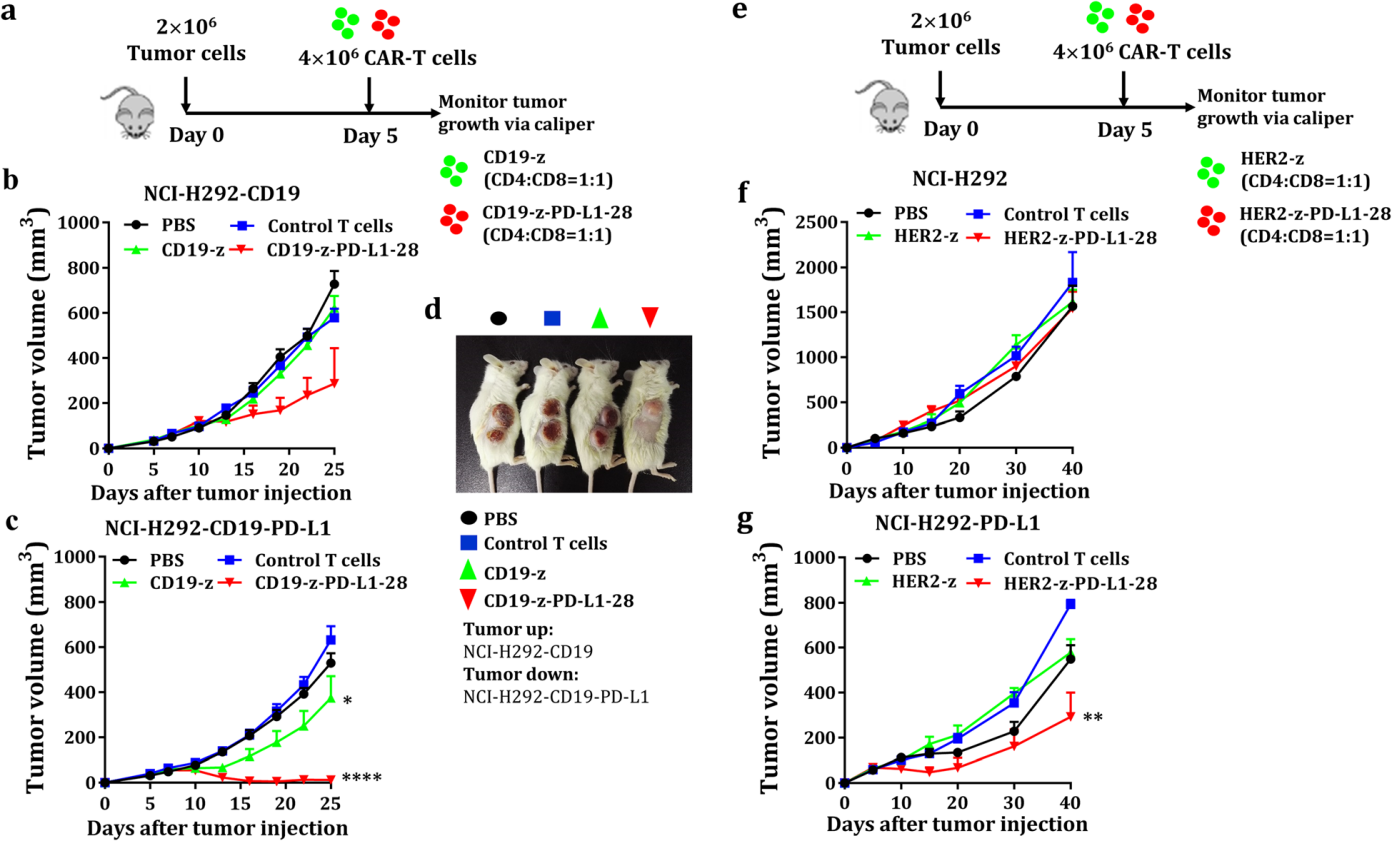
Tumoricidal effects of dual-targeted CAR-T cells in vivo. **a** Schematic diagram of the mouse treatment strategy. A total of 2 × 10^6^ NCI-H292-CD19 and 2 × 10^6^ NCI-H292-CD19-PD-L1 tumor cells were injected subcutaneously into the right flank of mice. A total of 2 × 10^6^ CD4^+^ T cells and 2 × 10^6^ CD8^+^ T cells expressing CD19-z or dual-targeted CAR were injected intravenously into these tumor-bearing mice. Tumor volume was monitored over 20 days after adoptive cell transfer. **b-c** Graphs presenting the tumor growth of CD19^+^ and CD19^+^PD-L1^+^ cells in mice treated with PBS, control T cells, CD19-z-engineered T cells and CD19-z-PD-L1–28-engineered T cells (*n* = 5 mice, error bars are the SEM, significance determined by one-way ANOVA, * *P* < 0.05, **** *P* < 0.0001 with respect to the control T cells treatment group). **d** Images showing tumor lesions in CD19^+^ (up) and CD19^+^PD-L1^+^ (down) NCI-H292 tumor-bearing mice were treated with PBS, control T cells and engineered T cells at 1 month after tumor cells inoculation. **e** NCI-H292 (2 × 10^6^) and NCI-H292-PD-L1 (2 × 10^6^) cells were injected subcutaneously into the right flank of mice. A total of 2 × 10^6^ CD4^+^ T cells and 2 × 10^6^ CD8^+^ T cells expressing HER2-z or HER2-z-PD-L1–28 were injected intravenously into these tumor-bearing mice. Tumor volume was monitored over 35 days after intravenous injection of the engineered T cells or control T cells. Where indicated, 2 × 10^6^ CD4^+^ T cells and 2 × 10^6^ CD8^+^ T cells transduced with HER2-z or HER2-z-PD-L1–28 were infused intravenously 5 days later. **f-g** Graphs presenting the tumor volume of HER2^+^ and HER2^+^PD-L1^+^ cells in mice treated with PBS, control T cells, HER2-z and HER2-z-PD-L1–28-engineered T cells (n = 5 mice, error bars are the SEM, significance determined by one-way ANOVA, ** *P* < 0.01 with respect to the control T cell treatment group)

To test whether the PD-L1 CCR CAR-T cells are functional in vivo, we determined the efficacy of dual-targeted CAR-T cells to eliminate tumor xenografts in the presence/absence of PD-L1 in humanized mice models. For this purpose, mice models bearing A549/NCI-H292 xenografts expressing a single tumor antigen (e.g. CD19 and HER2) or double antigens (TAAs and PD-L1) were used to evaluate antitumor efficacy of dual-targeted CAR-T cells. The tumor xenografts bearing a tumor antigen alone were used to mimic healthy normal tissues that may share an antigen with tumor cells to test on-target off-tumor toxicity of CAR-T cells. We found potent antitumor activities in those mice bearing CD19^+^PD-L1^+^ tumors treated with dual-targeted CAR-T cells (Fig. 6c) but much less in mice bearing CD19^+^ tumors (Fig. 6b), which was further demonstrated in A549 xenografts model (Additional file 5: Figure S5b-c). Interestingly, dual-targeted CAR-T cells are always more potent in controlling tumor xenografts than single-targeted CAR-T cells.

We next tested whether dual-targeted CAR-T cells could selectively inhibit PD-L1^+^ tumors but not normal tissues which usually expresses low levels of antigens in the absence of PD-L1. To mimic normal tissues expressing low levels of HER2, we chose NCI-H292 cells expressing low levels of HER2 (Additional file 2: Figure S2) to perform an in vivo cytotoxicity study. As expected, cytotoxic effect was not observed in the mice bearing NCI-H292 cells no matter treated with the first-generation HER2 CAR-T cells or dual-targeted CAR-T cells (Fig. 6f), However, the dual-targeted CAR-T cells could exert significantly suppress on HER2^+^PD-L1^+^ tumor xenografts (Fig. 6g), suggesting that dual-targeted CAR-T cells could selectively kill PD-L1^+^ tumor cells but not the normal tissues.

### The PD-L1 CCR improves antitumor activity of low-affinity CAR-T cells in vivo

**Fig. 7:**
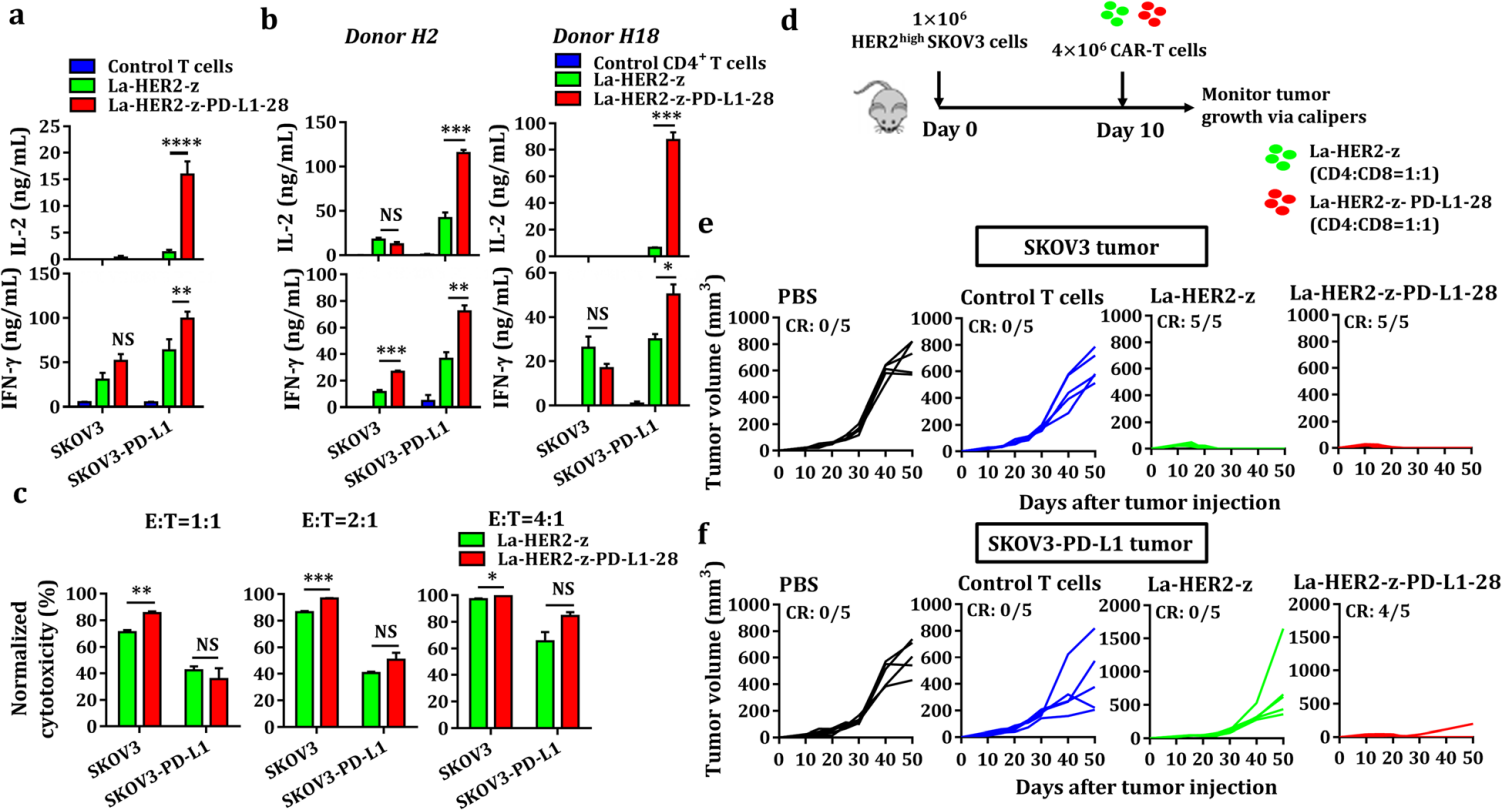
The antitumor activity of low-affinity dual-targeted CAR-T cells. **a** Cytokine secretion was assayed in supernatants from co-cultures of low-affinity HER2 CAR-T cells with or without the PD-L1 CCR. Bar charts show data from three healthy donors, which are represented as the mean ± SEM, for IL-2 and IFN-γ (NS, no significance; ** *P* < 0.01; **** *P* < 0.0001, analyzed using a paired Student’s t-test). **b** Cytokine release was assayed in supernatants from CAR-CD4^+^ T cells with or without the PD-L1 CCR. Each value represents the mean ± SEM of triplicates for IL-2 and IFN-γ (NS, no significance; * *P* < 0.05; ** *P* < 0.01; *** *P* < 0.001; analyzed using Student’s t-test). **c** Specific killing of PD-L1^−/+^ SKOV3 target cells by low-affinity HER2 CAR-T cells equipped with or without PD-L1 CCR was measured. The engineered T cells were co-cultured with PD-L1^+/−^ SKOV3 cells at the indicated E:T ratios after 18 h of incubation. Each value represents the mean ± SEM of technical triplicates (NS, no significance; * *P* < 0.05; ** *P* < 0.01; *** *P* < 0.001, analyzed using Student’s t-test). **d-f** The antitumor activity of low-affinity dual-targeted CAR-T cells in vivo. **d** Schematic diagram of T cells administration protocol. PD-L1^+/−^ SKOV3 cells were injected subcutaneously into the flank of mice on day 0. Ten days post-inoculation, the mice were randomized into four groups (n = 5 mice/group) and treated with 4 million control T cells, low-affinity HER2 CAR-T cells (CD4: CD8 = 1: 1), dual-targeted CAR-T cells (CD4: CD8 = 1: 1) or an equal volume of PBS. Tumor size was measured with calipers every 5 sssor 10 days. **e** Tumor growth curve for mice implanted with SKOV3 tumor xenografts. **f** Tumor size measurement for mice engrafted with PD-L1^+^SKOV3 tumor xenografts

A previous study showed that low-affinity HER2 CAR-T cells could control the growth of SKOV3 cells expressing high levels of HER2 but spare normal tissues expressing physiological HER2 levels, revealing that the application of low-affinity CAR is an effective and safe strategy to broaden the use of CAR-T immunotherapy [25]. PD-L1 is an immunosuppressive molecule that has been reported to be overexpressed in various solid tumors. Therefore, we further tested whether affinity-tuned HER2 CAR-T cells equipped with PD-L1 CCR could inhibit the growth of PD-L1^+^ solid tumors. Strikingly, low-affinity dual-targeted CAR-T cells secreted higher levels of IL-2 and IFN-γ in co-cultures of PD-L1^+^ SKOV3 cells than that in co-cultures of SKOV3 cells (Fig. 7a-b), indicating that PD-L1 CCR provided a strong costimulatory signal to antagonize PD-1/PD-L1 inhibition. Additionally, PD-L1 CCR slightly enhanced the cytolytic activity of low-affinity HER2 CAR-T cells against SKOV3 cells with or without PD-L1 (Fig. 7c). We next compared the antitumor efficacy of low-affinity dual-targeted CAR-T cells in the mice bearing SKOV3 and PD-L1^+^ SKOV3 tumor xenografts (Fig. 7d). Mice were treated with either low-affinity HER2 CAR-T cells or dual-targeted CAR-T cells exhibited complete remission of SKOV3 tumor xenografts (Fig. 7e). In contrast, uncontrolled tumor growth of PD-L1^+^ SKOV3 cells was observed in the mice treated with low-affinity HER2 CAR-T cells (Fig. 7f), indicating that PD-L1 significantly impaired the antitumor activity of low-affinity HER2 CAR-T cells. Importantly, low-affinity dual-targeted CAR-T cells still potently inhibited the growth of PD-L1^+^ SKOV3 tumor xenografts (Fig. 7f), implying that low-affinity CAR-T cells equipped with PD-L1 CCR could resist PD-L1 immunosuppression.

## Discussion

There exist many challenges in the clinical use of CAR-T cells to treat solid tumors, although CAR-T cells have shown good responses in hematological malignancies [1–5]. The following challenges need to be solved for the development of safe and effective CAR-T cells: tumor specific recognition, T cells trafficking and homing to tumor, T cells persistence in vivo, the reversion of immunosuppressive tumor microenvironment and the precise control of immune cells [26]. Among those challenges, on-target off-tumor toxicity caused by the TAAs that are typically used as target antigens needs to be reduced or avoided by improving precise tumor recognition. Depletion of normal B cells often occurs in CD19 CAR-T cells clinical studies and is tolerable with intravenous immunoglobulin therapy (IVIG) because CD19 is expressed on normal B cells [27]. However, there have been several clinical trials involving TAAs-targeting CAR-T cells within solid tumors, in which on-target off-tumor recognition of normal tissues has led to life-threatening adverse effects [6, 7]. Therefore, many researchers have attempted to improve the specificity of CAR-T cells, the combinatorial antigen recognition-based logic-gated CAR [8, 12–14], masked CAR [9], synNotch receptor [10, 28, 29] and SUPA CAR [11] were developed to reduce on-target off-tumor toxicity. Among these approaches, logic-gated CAR-engineered T cells (dual-targeted T cells) are fully activated only when both a suboptimal CAR and a CCR simultaneously recognize two TAAs on tumor cells, which markedly increases specificity and prevents normal tissue cells from attacking. Nevertheless, recent reports involving logic-gated CARs have shown that two different target antigens, CAR-targeted antigens and CCR-targeted antigens, must be carefully chosen to treat different solid tumors [8, 12–14], which is time consuming and laborious.

PD-L1, a key immune checkpoint molecule, was found to be overexpressed in a broad range of cancers [30], including breast, colon, gastric, lung, esophageal, ovarian, pancreatic, renal cell, and urothelial cancers. However, PD-L1 is also observed in activated cells [30], such as T cells, B cells, dendritic cells (DCs), macrophages [23, 24, 31–33], natural killer (NK) cells [34], and activated vascular endothelial cells (VECs) [35], which is not feasible for designing PD-L1-directed CARs but suitable for a universal target antigen for costimulatory domain-containing CCRs without TCR signaling. In this study, we confirmed that the CD28 costimulatory domain-containing PD-L1 CCR co-stimulated and promoted the activation of CAR-T cells, especially for CD4^+^ CAR-T cells. Enhanced cytokine release (e.g. IL-2) and proliferation were found in dual-targeted CAR-T cells expressing a suboptimal CD19/HER2 CAR and PD-L1 CCR, which is consistent with a previous study that showed the CD28 cytoplasmic domain induced a large amount of IL-2 that induced T cells expansion [36, 37]. The cytokine IL-2, a well-known T cell growth factor, plays an essential role in the immune responses [38], and increased IL-2 release caused by a costimulatory signal was also observed in reports involving logic-gated CARs [8, 12–14], which led to enhanced T cell activation and proliferation.

PD-L1 has been reported to be preferentially expressed on multiple solid tumor cells and several non-tumor cells, including DCs, macrophages and fibroblasts within tumor microenvironment (TME) [39]. Our data showed that dual-targeted CAR-T cells also responded to “by-standing” PD-L1-expressing host cells when they are simultaneously triggered by the cognate antigen expressed on tumor cells, indicating the great potential of the PD-L1 CCR in clinical applications, especially in contexts with PD-L1^−^ tumor cells but PD-L1-enriched TME-associated non-tumor cells [23, 24]. Meanwhile, dual-targeted CAR-T cells does not lyse PD-L1^+^ cells in the absence of TAAs on the same cells. These data suggest that dual-targeted CAR-T cells with PD-L1 CCR need the presence of both tumor-associated antigens and PD-L1 for optimal activation, which is usually the case for tumor cells and tumor microenvironment but unlikely for normal tissues or immune cells, thereby prevents those cells from optimal killing. In contrast, the single-targeted CAR-T cells effectively kill tumor cells expressing either a single antigen or double antigens in vitro [7, 8, 40], which poses the potential risk of an on-target off-tumor effect in high-affinity CAR-T cells. Indeed, our data further showed that low expression of antigens, as usually the case for normal tissues, failed to trigger the killing of dual-targeted CAR-T cells, and the presence of PD-L1 rescued their cytotoxicity, implicating that dual-targeted CAR-T are likely to be safe for normal tissues, but to be effective even when tumor cells express low level of tumor associated antigens.

To further improve the safety profile and minimize the risk of on-target off-tumor cytotoxicity of CAR-T cells, low affinity CAR-T cells may be used [25]. Unexpectedly, when using the aberrantly expressed tumor antigen (e.g. HER2) as a model, PD-L1 expression on tumor cells almost completely abolished the tumor suppression by low-affinity single-HER2 targeted CAR-T cells, PD-L1 CCR is able to fully reinvigorate the dual-targeted CAR-T cells to control tumor growth, suggesting that PD-L1 CCR could antagonized PD-L1 inhibition to improve the tumor-lytic activity of CAR-T cells whereas single-HER2-targeted CAR-T cells failed to do so.

In summary, the PD-L1 CCR may kill two birds with one stone: as a universal combinatorial antigen recognition target to reduce on-target off tumor cytotoxicity of engineered T cells in normal tissues but retain their effectiveness to kill tumor cells, and as a switch to turn “an immune brake” into “immune accelerator” and antagonize PD-L1 inhibition to improve the tumor-lytic activity of CAR-T cells [41, 42]. Considering the diversity of expression of PD-L1 in solid tumors and heterogeneity of TME, PD-L1 CCR should be further explored in clinical trial in different solid tumors.

## Conclusion

PD-L1 CCR, which includes the CD28 costimulatory domain, can be used to design logic-gated CARs and be likely to enhance the safety profile of CAR-T cells and improve their efficacy either when the low-affinity CAR is used or the tumor cells express low-level TAAs.

## Supplementary Information

**Additional file 1 Figure S1.** Design and characterization of the PD-L1 CAR. **a** The PD-L1 CAR consists of an extracellular humanized PD-L1-binding scFv and an intracellular human CD28, 4-1BB (also known as CD137), and CD3ζ signaling domain. SP, signal peptide; Flag, DYKDDDDK epitope; HTM, hinger and transmembrane domain. **b** The generation of PD-L1-expressing tumor cells. Wild-type K562 and A549 tumor cells were transduced with pseudotyped lentivirus encoding both PD-L1 and puromycin. PD-L1-expressing tumor cells were first selected and enriched by adding puromycin to the culture medium and further sorted by a BD FACSAria. **c** The expression of PD-L1 CAR was determined by FACS using a PE-conjugated anti-DYKDDDDK antibody for untransduced Jurkat T cells and engineered Jurkat T cells. **d** CFSE-labeled untransduced or PD-L1 CAR-expressing Jurkat T cells were cocultured with PD-L1-positive/negative-K562 tumor cells labeled with eFluor 670 at RT for 1 h. The percentage of cell aggregates is quantified in the upper right quadrant of each 2D flow cytometry dot plot. **e** The levels of IL-2 produced by untransduced or PD-L1 CAR-engineered Jurkat T cells were measured by ELISA after 24 h incubation at an E:T ratio of 1:1 (K562) or 3:1 (A549). The results are reported as the mean ± SEM for three independent experiments, * *P* < 0.05 with respect to coculture with PD-L1-negative K562 or A549 cells, analyzed using Student’s t-test.

**Additional file 2 Figure S2.** The differential expression of CD19, HER2 and PD-L1 on tumor cells. A total of 5 × 10^5^ tumor cells were harvested and washed twice with FACS buffer. Then, tumor cells were stained with 2 μg/mL trastuzumab previously prepared in our laboratory at 4 °C for 30 min, washed twice with FACS buffer, further stained with 0.5 μL of PE-conjugated anti-human IgG Fc at 4 °C for 30 min, washed twice with FACS buffer, and resuspended in FACS buffer to detect HER2. Tumor cells stained with PE-conjugated anti-human IgG Fc served as a blank control. Similarly, 5 × 10^5^ tumor cells were harvested and washed twice with FACS buffer, stained with 0.5 μL of APC/Cy7-conjugated mouse anti-human CD19 or APC-conjugated mouse anti-human PD-L1 at 4 °C for 30 min, washed twice with FACS buffer, and then resuspended in FACS buffer for assessment. The unstained tumor cells served as a blank control.

**Additional file 3 Figure S3.** The surface expression of the HER2 CAR and PD-L1 CCR on T cells. **a** Schematic representation of the HER2 CAR and PD-L1 CCR constructs. The HER2 CAR (HER2-z) was generated by using the first generation of the CAR that fuses the HER2-specific scFv to the human CD8 hinger and transmembrane domain, followed by the CD3ζ cytosolic signaling domain. HER2-z-PD-L1–28 was generated by linking HER2-z to the PD-L1 CCR, which was generated by fusing a humanized PD-L1-binding scFv to the hinger, transmembrane and intracellular signaling domains of human CD28, followed by the self-cleaving T2A peptide sequence. **b** A total of 5 × 10^5^ T cells were harvested and washed twice with FACS buffer, stained with 0.5 μL of Alexa Fluor 647-conjugated anti-Myc tag and 0.5 μL of PE-conjugated anti-DYKDDDDK at 4 °C for 30 min, washed twice with FACS buffer, and resuspended in FACS buffer to detect the HER2 CAR and PD-L1 CCR. The percentage of positive cells was quantified in the upper right quadrant of each 2D flow cytometry contour diagram.

**Additional file 4 Figure S4.** Cytotoxicity of engineered T cells against cancer cells. **a-b** Untransduced, CD19-z, CD19-z-PD-L1–28-engineered T cells were used in coculture with PD-L1^+/−^ A549-CD19, NCI-H292-CD19 or K562-PD-L1 cells at the indicated E:T ratios for 18 h. The results shown are the mean ± SEM for three healthy donors. * *P* < 0.05, ** *P* < 0.01, *** *P* < 0.001, **** *P* < 0.0001 with respect to coculture with CAR-T cells without the PD-L1 CCR, analyzed using a paired Student’s t-test. **c-d** Untransduced, CD19-z, CD19-z-PD-L1–28-engineered T cells were used in coculture with PD-L1^+^ K562 and A549-CD19 or NCI-H292-CD19 cells at the indicated E:T ratios for 18 h. Each value shown is the mean ± SEM of triplicates.

**Additional file 5 Figure S5.** Therapeutic efficacy of PD-L1 CCR-engineered CD19 CAR-T cells in vivo. **a** Schematic diagram of the mouse treatment strategy. A total of 5 × 10^6^ CD19^+^ and 5 × 10^6^ CD19^+^PD-L1^+^ A549 tumor cells were injected subcutaneously into the same right flank of B-NDG mice. A total of 2 × 10^6^ CD4^+^ and 2 × 10^6^ CD8^+^ T cells expressing CD19-z or CD19-z-PD-L1–28 were injected intravenously into these tumor-bearing mice. Tumor volume was monitored over 25 days after intravenous injection of engineered T cells or untransduced T cells (control T cells). **b-c** Graphs presenting CD19^+^ (**b**) and CD19^+^PD-L1^+^ (**c**) tumor volumes for mice treated with PBS, untransduced T cells, CD19-z-expressing T cells and CD19-z-PD-L1–28-engineered T cells.

## Data Availability

The datasets used and/or analyzed during the current study are available from the corresponding author on reasonable request.

## Abbreviations

CAR-T cells: Chimeric antigen receptor T cells
TAAs: Tumor-associated antigens
CCR: Chimeric costimulatory receptor
PD-L1: Programmed death-ligand 1
ELISA: Enzyme-linked immunosorbent assay
CBA: Cytometric bead array
FDA: Food and Drug Administration
CAIX: Carbonic anhydrase IX
HER2: Human epidermal growth factor receptor 2
MMPs: Matrix metalloproteinases
RPMI 1640: Roswell Park Memorial Institute 1640
DMEM: Dulbecco’s modified Eagle’s medium
FBS: Fetal bovine serum
ScFv: Single-chain variable fragment
SP: Signal peptide
PBMCs: Peripheral blood mononuclear cells
TCM: T cell growth medium
CFSE: Carboxyfluorescein diacetate succinimidyl ester
RT: Room temperature
FACS: Fluorescence-activated cell sorting
E:T ratio: Effector: target ratio
IACUC: Institutional Animal Care and Use Committee
SEM: Standard error of the mean
ANOVA: Analysis of variance
TSAs: Tumor-specific antigens
IVIG: Intravenous immunoglobulin
DCs: Dendritic cells
NK: Natural killer
TME: Tumor microenvironment
