# Supplementary material for: PD-L1 chimeric costimulatory receptor improves the efficacy of CAR-T cells for PD-L1-positive solid tumors and reduces toxicity in vivo
Source: Biomark Res. 2020 Nov 2;8:57. doi: 10.1186/s40364-020-00237-w (PMC7607631; doi:10.1186/s40364-020-00237-w)
Supplement: Supplementary file 1 — Additional file 1 Figure S1. Design and characterization of the PD-L1 CAR. a The PD-L1 CAR consists of an extracellular humanized PD-L1-binding scFv and an intracellular human CD28, 4-1BB (also known as CD137), and CD3ζ signaling domain. SP, signal peptide; Flag, DYKDDDDK epitope; HTM, hinger and transmembrane domain. b The generation of PD-L1-expressing tumor cells. Wild-type K562 and A549 tumor cells were transduced with pseudotyped lentivirus encoding both PD-L1 and puromycin. PD-L1-expressing tumor cells were first selected and enriched by adding puromycin to the culture medium and further sorted by a BD FACSAria. c The expression of PD-L1 CAR was determined by FACS using a PE-conjugated anti-DYKDDDDK antibody for untransduced Jurkat T cells and engineered Jurkat T cells. d CFSE-labeled untransduced or PD-L1 CAR-expressing Jurkat T cells were cocultured with PD-L1-positive/negative-K562 tumor cells labeled with eFluor 670 at RT for 1 h. The percentage of cell aggregates is quantified in the upper right quadrant of each 2D flow cytometry dot plot. e The levels of IL-2 produced by untransduced or PD-L1 CAR-engineered Jurkat T cells were measured by ELISA after 24 h incubation at an E:T ratio of 1:1 (K562) or 3:1 (A549). The results are reported as the mean ± SEM for three independent experiments, * P < 0.05 with respect to coculture with PD-L1-negative K562 or A549 cells, analyzed using Student’s t-test. [file 40364_2020_237_MOESM1_ESM.docx]

**
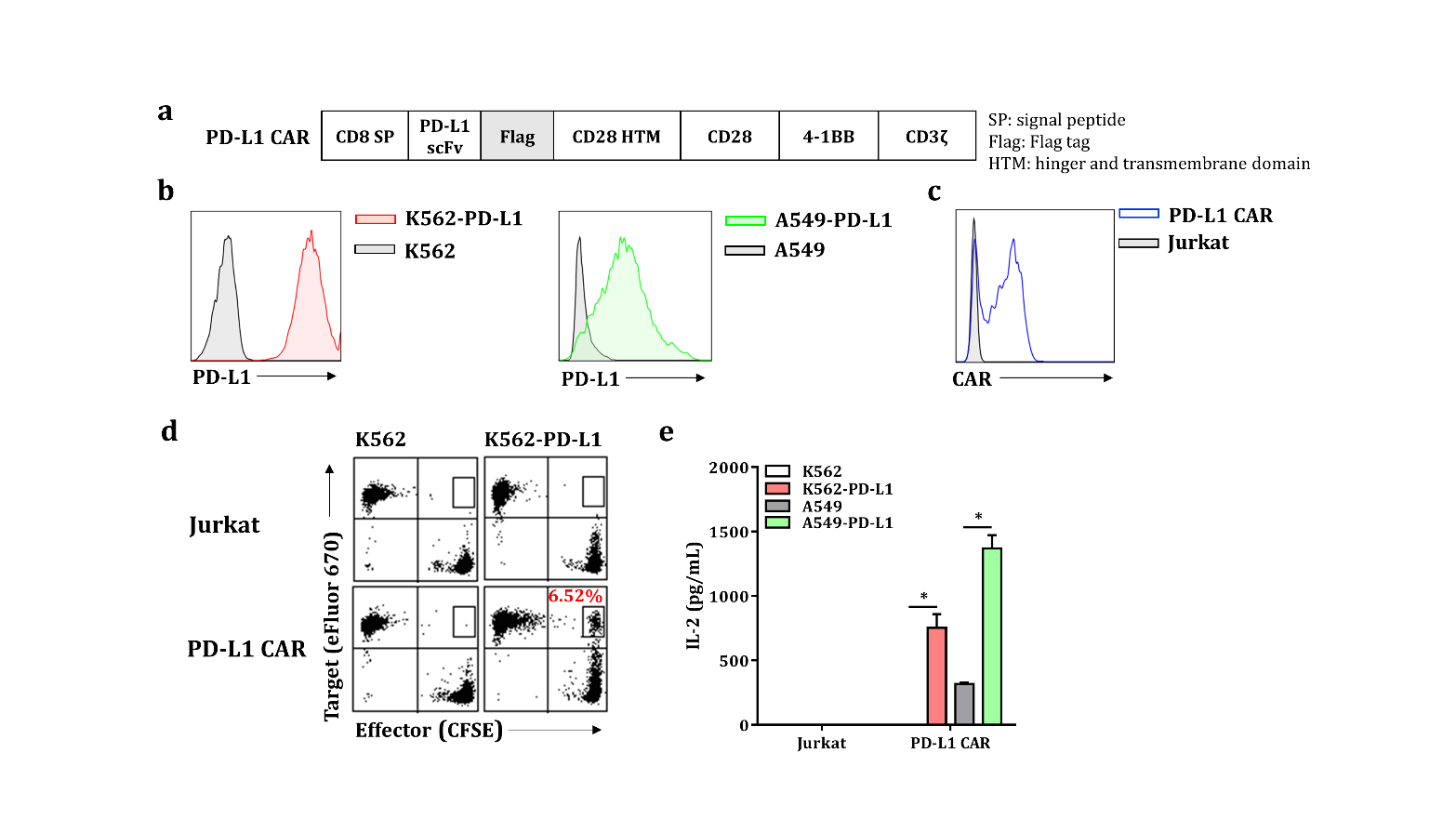
**

**Additional file 1: Figure S1.** Design and characterization of the PD-L1 CAR. **a** The PD-L1 CAR consists of an extracellular humanized PD-L1-binding scFv and an intracellular human CD28, 4-1BB (also known as CD137), and CD3ζ signaling domain. SP, signal peptide; Flag, DYKDDDDK epitope; HTM, hinger and transmembrane domain. **b** The generation of PD-L1-expressing tumor cells. Wild-type K562 and A549 tumor cells were transduced with pseudotyped lentivirus encoding both PD-L1 and puromycin. PD-L1-expressing tumor cells were first selected and enriched by adding puromycin to the culture medium and further sorted by a BD FACSAria. **c** The expression of PD-L1 CAR was determined by FACS using a PE-conjugated anti-DYKDDDDK antibody for untransduced Jurkat T cells and engineered Jurkat T cells. **d** CFSE-labeled untransduced or PD-L1 CAR-expressing Jurkat T cells were cocultured with PD-L1-positive/negative-K562 tumor cells labeled with eFluor 670 at RT for 1 h. The percentage of cell aggregates is quantified in the upper right quadrant of each 2D flow cytometry dot plot. **e** The levels of IL-2 produced by untransduced or PD-L1 CAR-engineered Jurkat T cells were measured by ELISA after 24 h incubation at an E:T ratio of 1:1 (K562) or 3:1 (A549). The results are reported as the mean ± SEM for three independent experiments, * *P* < 0.05 with respect to coculture with PD-L1-negative K562 or A549 cells, analyzed using Student’s t-test.
