# Supplementary material for: PD-L1 chimeric costimulatory receptor improves the efficacy of CAR-T cells for PD-L1-positive solid tumors and reduces toxicity in vivo
Source: Biomark Res. 2020 Nov 2;8:57. doi: 10.1186/s40364-020-00237-w (PMC7607631; doi:10.1186/s40364-020-00237-w)
Supplement: Supplementary file 2 — Additional file 2 Figure S2. The differential expression of CD19, HER2 and PD-L1 on tumor cells. A total of 5 × 10^5^ tumor cells were harvested and washed twice with FACS buffer. Then, tumor cells were stained with 2 μg/mL trastuzumab previously prepared in our laboratory at 4 °C for 30 min, washed twice with FACS buffer, further stained with 0.5 μL of PE-conjugated anti-human IgG Fc at 4 °C for 30 min, washed twice with FACS buffer, and resuspended in FACS buffer to detect HER2. Tumor cells stained with PE-conjugated anti-human IgG Fc served as a blank control. Similarly, 5 × 10^5^ tumor cells were harvested and washed twice with FACS buffer, stained with 0.5 μL of APC/Cy7-conjugated mouse anti-human CD19 or APC-conjugated mouse anti-human PD-L1 at 4 °C for 30 min, washed twice with FACS buffer, and then resuspended in FACS buffer for assessment. The unstained tumor cells served as a blank control. [file 40364_2020_237_MOESM2_ESM.docx]

**
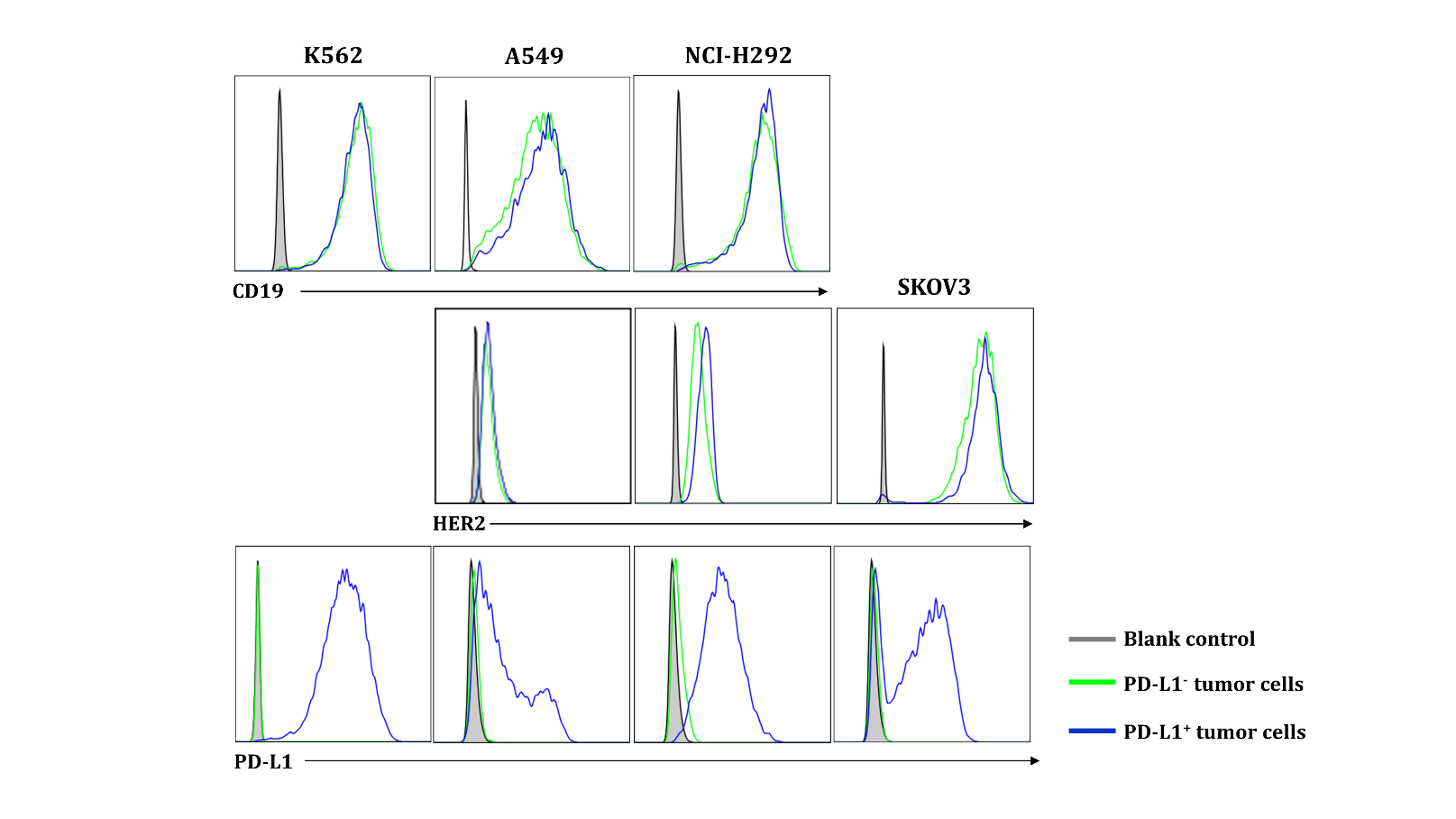
**

**Additional file 2: Figure S2.** The differential expression of CD19, HER2 and PD-L1 on tumor cells. A total of 5×10^5^ tumor cells were harvested and washed twice with FACS buffer. Then, tumor cells were stained with 2 μg/mL trastuzumab previously prepared in our laboratory at 4°C for 30 min, washed twice with FACS buffer, further stained with 0.5 μL of PE-conjugated anti-human IgG Fc at 4°C for 30 min, washed twice with FACS buffer, and resuspended in FACS buffer to detect HER2. Tumor cells stained with PE-conjugated anti-human IgG Fc served as a blank control. Similarly, 5×10^5^ tumor cells were harvested and washed twice with FACS buffer, stained with 0.5 μL of APC/Cy7-conjugated mouse anti-human CD19 or APC-conjugated mouse anti-human PD-L1 at 4°C for 30 min, washed twice with FACS buffer, and then resuspended in FACS buffer for assessment. The unstained tumor cells served as a blank control.
