# Supplementary material for: PD-L1 chimeric costimulatory receptor improves the efficacy of CAR-T cells for PD-L1-positive solid tumors and reduces toxicity in vivo
Source: Biomark Res. 2020 Nov 2;8:57. doi: 10.1186/s40364-020-00237-w (PMC7607631; doi:10.1186/s40364-020-00237-w)
Supplement: Supplementary file 3 — Additional file 3 Figure S3. The surface expression of the HER2 CAR and PD-L1 CCR on T cells. a Schematic representation of the HER2 CAR and PD-L1 CCR constructs. The HER2 CAR (HER2-z) was generated by using the first generation of the CAR that fuses the HER2-specific scFv to the human CD8 hinger and transmembrane domain, followed by the CD3ζ cytosolic signaling domain. HER2-z-PD-L1–28 was generated by linking HER2-z to the PD-L1 CCR, which was generated by fusing a humanized PD-L1-binding scFv to the hinger, transmembrane and intracellular signaling domains of human CD28, followed by the self-cleaving T2A peptide sequence. b A total of 5 × 10^5^ T cells were harvested and washed twice with FACS buffer, stained with 0.5 μL of Alexa Fluor 647-conjugated anti-Myc tag and 0.5 μL of PE-conjugated anti-DYKDDDDK at 4 °C for 30 min, washed twice with FACS buffer, and resuspended in FACS buffer to detect the HER2 CAR and PD-L1 CCR. The percentage of positive cells was quantified in the upper right quadrant of each 2D flow cytometry contour diagram. [file 40364_2020_237_MOESM3_ESM.docx]

**
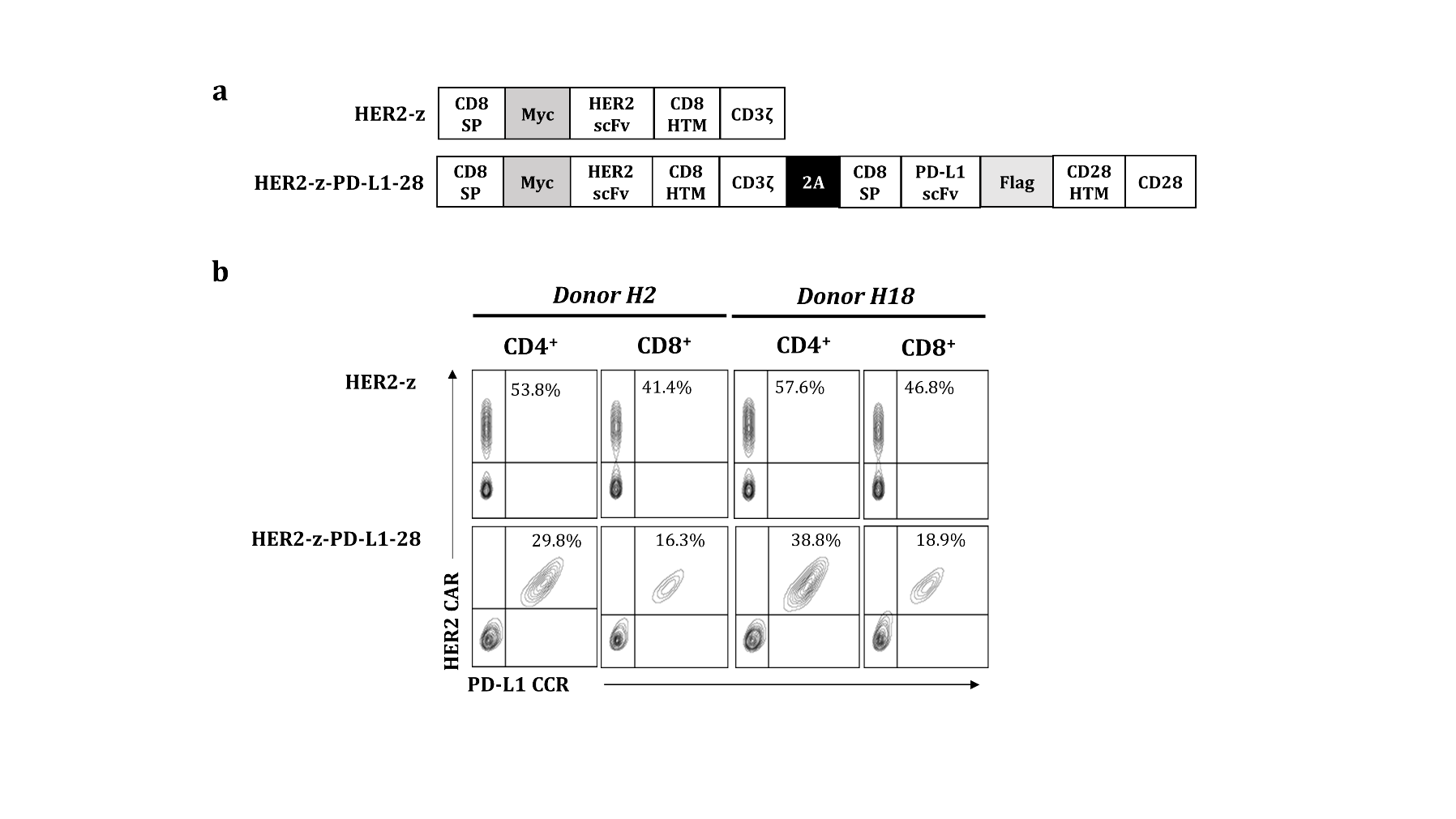
**

**Additional file 3: Figure S3.** The surface expression of the HER2 CAR and PD-L1 CCR on T cells. **a** Schematic representation of the HER2 CAR and PD-L1 CCR constructs. The HER2 CAR (HER2-z) was generated by using the first generation of the CAR that fuses the HER2-specific scFv to the human CD8 hinger and transmembrane domain, followed by the CD3ζ cytosolic signaling domain. HER2-z-PD-L1-28 was generated by linking HER2-z to the PD-L1 CCR, which was generated by fusing a humanized PD-L1-binding scFv to the hinger, transmembrane and intracellular signaling domains of human CD28, followed by the self-cleaving T2A peptide sequence. **b** A total of 5×10^5^ T cells were harvested and washed twice with FACS buffer, stained with 0.5 μL of Alexa Fluor 647-conjugated anti-Myc tag and 0.5 μL of PE-conjugated anti-DYKDDDDK at 4°C for 30 min, washed twice with FACS buffer, and resuspended in FACS buffer to detect the HER2 CAR and PD-L1 CCR. The percentage of positive cells was quantified in the upper right quadrant of each 2D flow cytometry contour diagram.
