# Supplementary material for: PD-L1 chimeric costimulatory receptor improves the efficacy of CAR-T cells for PD-L1-positive solid tumors and reduces toxicity in vivo
Source: Biomark Res. 2020 Nov 2;8:57. doi: 10.1186/s40364-020-00237-w (PMC7607631; doi:10.1186/s40364-020-00237-w)
Supplement: Supplementary file 4 — Additional file 4 Figure S4. Cytotoxicity of engineered T cells against cancer cells. a-b Untransduced, CD19-z, CD19-z-PD-L1–28-engineered T cells were used in coculture with PD-L1+/− A549-CD19, NCI-H292-CD19 or K562-PD-L1 cells at the indicated E:T ratios for 18 h. The results shown are the mean ± SEM for three healthy donors. * P < 0.05, ** P < 0.01, *** P < 0.001, **** P < 0.0001 with respect to coculture with CAR-T cells without the PD-L1 CCR, analyzed using a paired Student’s t-test. c-d Untransduced, CD19-z, CD19-z-PD-L1–28-engineered T cells were used in coculture with PD-L1+ K562 and A549-CD19 or NCI-H292-CD19 cells at the indicated E:T ratios for 18 h. Each value shown is the mean ± SEM of triplicates. [file 40364_2020_237_MOESM4_ESM.docx]

**
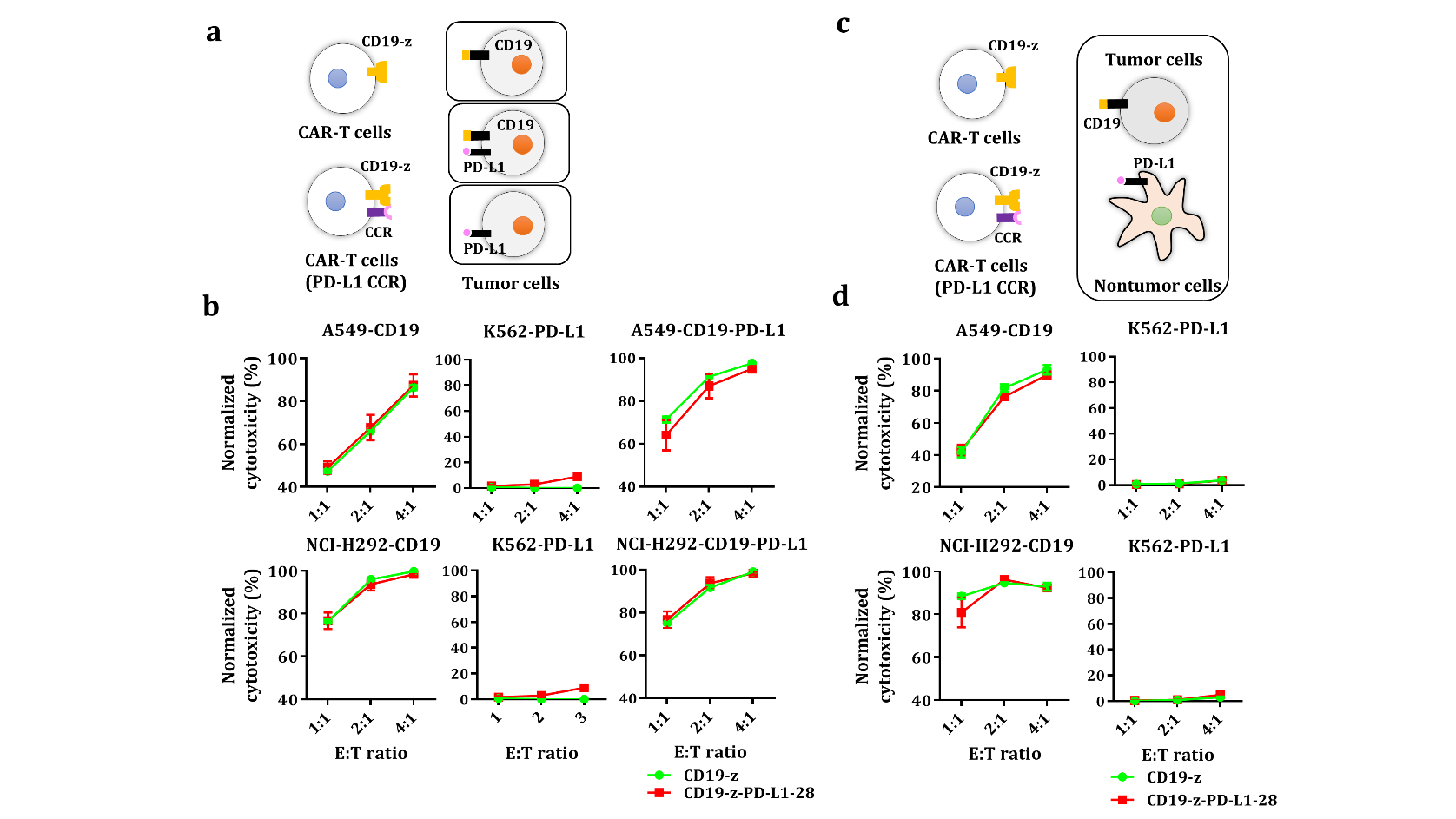
**

**Additional file 4: Figure S4.** Cytotoxicity of engineered T cells against cancer cells. **a-b** Untransduced, CD19-z, CD19-z-PD-L1-28-engineered T cells were used in coculture with PD-L1^+/-^ A549-CD19, NCI-H292-CD19 or K562-PD-L1 cells at the indicated E:T ratios for 18 h. The results shown are the mean ± SEM for three healthy donors. * P < 0.05, ** P < 0.01, *** P < 0.001, **** P < 0.0001 with respect to coculture with CAR-T cells without the PD-L1 CCR, analyzed using a paired Student’s t-test. **c-d** Untransduced, CD19-z, CD19-z-PD-L1-28-engineered T cells were used in coculture with PD-L1^+^ K562 and A549-CD19 or NCI-H292-CD19 cells at the indicated E:T ratios for 18 h. Each value shown is the mean ± SEM of triplicates.
