# Supplementary material for: PD-L1 chimeric costimulatory receptor improves the efficacy of CAR-T cells for PD-L1-positive solid tumors and reduces toxicity in vivo
Source: Biomark Res. 2020 Nov 2;8:57. doi: 10.1186/s40364-020-00237-w (PMC7607631; doi:10.1186/s40364-020-00237-w)
Supplement: Supplementary file 5 — Additional file 5 Figure S5. Therapeutic efficacy of PD-L1 CCR-engineered CD19 CAR-T cells in vivo. a Schematic diagram of the mouse treatment strategy. A total of 5 × 10^6^ CD19+ and 5 × 10^6^ CD19+PD-L1+ A549 tumor cells were injected subcutaneously into the same right flank of B-NDG mice. A total of 2 × 10^6^ CD4+ and 2 × 10^6^ CD8+ T cells expressing CD19-z or CD19-z-PD-L1–28 were injected intravenously into these tumor-bearing mice. Tumor volume was monitored over 25 days after intravenous injection of engineered T cells or untransduced T cells (control T cells). b-c Graphs presenting CD19+ (b) and CD19+PD-L1+ (c) tumor volumes for mice treated with PBS, untransduced T cells, CD19-z-expressing T cells and CD19-z-PD-L1–28-engineered T cells. [file 40364_2020_237_MOESM5_ESM.docx]

**
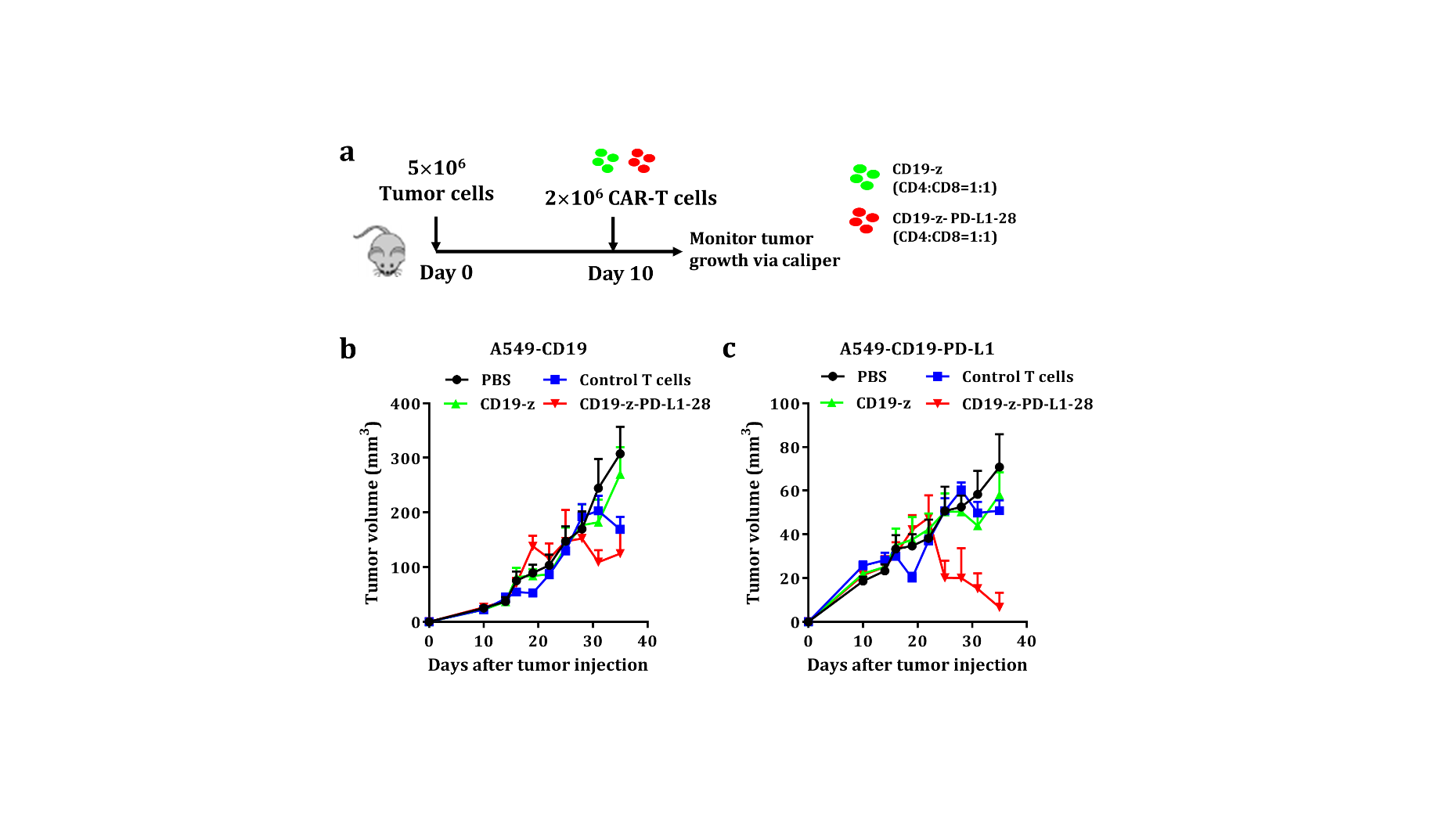
**

**Additional file 5: Figure S5.** Therapeutic efficacy of PD-L1 CCR-engineered CD19 CAR-T cells *in vivo*. **a** Schematic diagram of the mouse treatment strategy. A total of 5×10^6^ CD19^+^ and 5×10^6^ CD19^+^PD-L1^+^ A549 tumor cells were injected subcutaneously into the same right flank of B-NDG mice. A total of 2×10^6^ CD4^+^ and 2×10^6^ CD8^+^ T cells expressing CD19-z or CD19-z-PD-L1-28 were injected intravenously into these tumor-bearing mice. Tumor volume was monitored over 25 days after intravenous injection of engineered T cells or untransduced T cells (control T cells). **b-c** Graphs presenting CD19^+^ (**b**) and CD19^+^PD-L1^+^ (**c**) tumor volumes for mice treated with PBS, untransduced T cells, CD19-z-expressing T cells and CD19-z-PD-L1-28-engineered T cells.
